# Molecular Events Controlling Cessation of Trunk Neural Crest Migration and Onset of Differentiation

**DOI:** 10.3389/fcell.2020.00199

**Published:** 2020-04-02

**Authors:** Vivian M. Lee, Sergio Hernandez, Belle Giang, Chris Chabot, Jazmir Hernandez, Maria Elena de Bellard

**Affiliations:** ^1^Universal Cells Inc., Seattle, WA, United States; ^2^Biology Department, California State University Northridge, Northridge, CA, United States; ^3^Moorpark College, Moorpark, CA, United States

**Keywords:** neural crest, migration, ganglion formation, condensation, trunk, dorsal root ganglia

## Abstract

Neural crest cells (NCC) migrate extensively in vertebrate embryos to populate diverse derivatives including ganglia of the peripheral nervous system. Little is known about the molecular mechanisms that lead migrating trunk NCC to settle at selected sites in the embryo, ceasing their migration and initiating differentiation programs. To identify candidate genes involved in these processes, we profiled genes up-regulated in purified post-migratory compared with migratory NCC using a staged, macroarrayed cDNA library. A secondary screen of *in situ* hybridization revealed that many genes are specifically enhanced in neural crest-derived ganglia, including macrophage migration inhibitory factor (MIF), a ligand for CXCR4 receptor. Through *in vivo* and *in vitro* assays, we found that MIF functions as a potent chemoattractant for NCC. These results provide a molecular profile of genes expressed concomitant with gangliogenesis, thus, offering new markers and potential regulatory candidates involved in cessation of migration and onset of differentiation.

## Introduction

Neural crest cells (NCC) are the most migratory cell types in the developing vertebrate embryo. Starting from the dorsal neural tube, these cells undergo an epithelial to mesenchymal transition (EMT) exiting the dorsal neural tube (NT) as individual cells. They then migrate extensively to diverse areas in the embryo giving rise to a wide range of different cell types. For example, the sensory neurons begin as NCC and terminally reside in the dorsal root ganglion (DRG) along the trunk (Raible and Ungos, 2006).

The process of NCC migration is a fascinating one. First, NCC migration is fast, collective and follows stereotypical pathways that make it a unique and excellent model to study collective cell motility under different lenses (Gitler et al., 2002; Bronner and LaBonne, 2012; Szabó and Mayor, 2018). Second, it is an excellent model for cancer metastasis given the similarities in genes expressed during NCC EMT and cancer EMT (Thiery et al., 2009; Strobl-Mazzulla and Bronner, 2012). Third, these highly migratory cells need to stop along their pathways to begin forming different embryo structures: from sensory ganglia, to cornea, to skin melanocytes, to adrenal glands, to peripheral nerve sheaths (Le Douarin, 2008). This last step has been likened to Mesenchymal-to-Epithelial Transition or MET that occurs in cancer cells (Wells et al., 2008; Acloque et al., 2009; Lim and Thiery, 2012). However, NCC do not become epithelial cells upon condensing into ganglions, and so this transition is more accurately named Mesenchymal-to-Ganglionic-Transition (MGT).

While a good deal is known about the signals and cellular changes that govern NCC EMT and the differentiation process that leads them to transform into neurons, glia, melanocytes, etc. (Le Douarin and Dupin, 2003; Tang et al., 2019), surprisingly very little is known about the molecular changes that accompany cessation of NCCs migration and their specific localization into discrete derivatives groups. How do these cells recognize that they have reached their proper destination, stopped migrating and must transform into a differentiated, functional cells? (Achilleos and Trainor, 2012). Recent work from Kulesa’s and Bronner’s labs have looked at the molecular signatures of migrating trunk NCC (tNCC) (Betancur et al., 2014; Simoes-Costa et al., 2014; McLennan et al., 2015; Morrison et al., 2017). Thus, we now know the main players that govern NCC identity and fate, also referred as gene regulatory network (GRN) (Sauka-Spengler and Bronner-Fraser, 2006; Betancur et al., 2010). We also know which set of genes migrating NCC use when reaching their targets: GDNF, PDGF, VEGF, and SDF1 (McLennan et al., 2015; Morrison et al., 2017). While these two mechanisms are fundamental in our understanding of the nature of NCC, we still lack a view at the reversal of the events mediating the end of NCC migration, which we now refer to as MGT. Some light may come from cancer studies looking at MET transition. One study looking at stem cells found that BMP signaling promotes reprogramming of stem cells toward an epithelial phenotype by increasing expression of E-Cadherin (CDH1), claudins, occluding, etc. (Samavarchi-Tehrani et al., 2010; Takaishi et al., 2016). Another recent study shows that the tumor suppressor OVOL2 can induce MET in fibroblasts (Watanabe et al., 2019). If we will guide ourselves by the GRN recently updated by Simoes-Costa (Simões-Costa and Bronner, 2015), we would expect to find in our screen the following genes: TFAP (activator of neural plate border specifier), MSX1 (a transcription factor that is required for NCC induction), WNT (another required gene for NCC specification in the neural plate border) or GATA2/3 (this is transcription factor is involved in peripheral neurons development (Tsarovina et al., 2010).

In order to examine the overall molecular changes that occur at post-migratory stages of trunk NCC (tNCC) we took a genomics approach and generated a profile of the molecular changes that accompany cessation of migration and aggregation into DRG. These changes can be clearly separated by isolating tNCC from two stages in chicken embryo: during peak of tNCC migration (HH16) and when the future DRG tNCC begin to stop next to the neural tube (HH19) (Giovannone et al., 2015). We generated a tNCC macroarray library of purified chicken tNCC at these two stages and then compared their gene expression by probing an arrayed cDNA library. Our findings offer the first comprehensive profile of molecular changes associated with late stages of tNCC migration and provide numerous candidate genes for future functional analysis. One gene of interest based on our differential array was the CXCR4 and its ligand Macrophage inhibitory factor (MIF). We corroborated that MIF is a functional signaling chemokine for tNCC by chemotaxis and chemokinetic experiments, and found out that MIF is a chemoattractant for trunk NCC.

## Materials and Methods

### Avian Embryos

Fertilized quail (Coturnix coturnix japonica) and chick (Gallus gallus domesticus, White Leghorn and Rhode Island Red) eggs were obtained from commercial sources and incubated at 38°C in a humidified atmosphere.

### Library Synthesis

The HH16-19 chick bacterial cDNA library was synthesized using the Invitrogen SuperScript cDNA Synthesis and Plasmid Cloning kit. HH16-19 embryos were collected and poly-A RNA isolated. First strand cDNA was prepared using an oligo-dT/*Not*I primer/adapter and SuperScript reverse transcriptase. Second strand cDNA was prepared using standard Gubler and Hoffman nick translational replacement of the mRNA. *Sal*I linkers were ligated, and the cDNA was directionally cloned into the pCMV-Sport6 vector that was included in the kit. The library had a complexity of 1.5 × 10^8^ clones/μg with an average insert size of 2 kb.

### Array Production

The library was arrayed and spotted using the Genomix Q-bot in the Genomics Technology Facility at Caltech. 10 filters, comprising >180,000 different clones, were spotted. This number was predicted to include most genes in the library at least once. About 2 ng of DNA were reproducibly deposited at each spot.

Three biological replicates of total RNA extracted from each stage of purified NCC were analyzed with Agilent micro analyzer and the integrity of total RNA by the ratio to 18s and 28s rRNA. The macroarray analysis was performed by Analysis expression and analyzed the normalization of the macroarray data. The gene expression level in each comparison were the mean value of the three replicates. The twofold up-regulated or down regulated genes with *P*-value less than 0.01 were chosen as differential expressed genes. The differential expressed genes were used for further GO and Ingenuity analysis.

### Isolation and Purification of Trunk Neural Crest Cells From Avian Embryos

Explants from somite levels 11–22 (HH16 and HH19 trunks) were isolated from chicken embryos and treated with Dispase to facilitate the removal of ectoderm and notochord (which also expresses HNK1). The remaining tissues were triturated into single cells with a fire-polished pipette. The cell suspension was incubated with HNK1 for 10–15 min and then a rat anti-mouse IgM conjugated to magnetic microbeads (Miltenyi Biotech) was added to the cells according to manufacturer’s instructions followed by a rhodamine red-X conjugated donkey anti-mouse IgM antibody (Jackson Immuno Research). Samples from each purification experiments were counted before and after purification on a hemocytometer to estimate yield and subjected to cytospin to visualize the purity of HNK1 + cells under a fluorescent microscope. About 500,000 cells could be recovered from 40 to 50 HH19 embryos and 100,000 cells from 90 to 100 HH16 trunks.

In order to corroborate our trunk analysis, we also isolated pure cranial NCC from HH9 chicken embryo. Here the embryo heads were cut in the boundary of hindbrain and midbrain. After removing neural tube and notochords the head tissue was digested with Dispase, mechanically triturated into single cell, and then purified with MACS method (70–150 embryos were pooled each time). The single cranial NCC were first incubated with mouse anti-chicken HNK1-IgM antibody. Unbound antibody was removed by 3 washes in PBS. Then the cells were incubated with anti-mouse IgM-beads antibody (Miltenyl Biotech or Dynal Lab) at 4°C for 10 min and further incubated with anti-mouse IgM-RRX antibody for further evaluation of the purity of the purified NCC. After 5 times washes in PBS, the cells were passed through MACS column and the HNK1-positive cells were kept in the column for further elution with degassed PBS buffer. The purified cells were then lysed with Ambion lysis buffer and keep in −80°C for extraction of RNA. The purity of the cells were evaluated by using cytospin centrifuge to spread the cells on the slide and the percentage of the HNK1 positive cells were calculated by counting five 40X magnification field. Only NCC that have the purity more than 95% were used for the HH9 macroarray analysis.

### Total RNA Isolation

Purified HNK1 + cells from each experiment were collected immediately using the Ambion RNA lysis buffer and store at −80°C until use. Total RNA was isolated using the Ambion RNAqueous kit according to manufacturer’s protocol. RNA samples were analyzed on a MOPS gel and spectrophotometer to determine their integrity and quantity.

The total RNA was extracted using Qiagen Kit. The integrity of the total RNA was analyzed with Angialent microanalizer before labeling. The total RNA was used to macroarray analysis with Affymetrix gallus genome oligonucleotide macroarray was analyzed by Analysis Expression Company. To identify the migration and differentiation related genes, we compared the HH19 tNCC with HH16 tNCC to find the differential expressed genes that contributed to the migration and differentiation of tNCC.

### Functional Annotation and Bioinformatics Analysis

Differentially expressed genes (twofold change, *p* < 0.05) were analyzed according to predefined pathways of or functional categories annotated by KEGG (Kanehisa and Goto, 2000) Biocartia (Biocartia Pathways)^1^ and GO using DAVID bioinformatics resources functional annotation tool.^2^ For an overrepresented GO Biocartia or KEGG pathway, a cut off *p*-value of 0.1 was chosen. It should be noted that one gene can participate in more than one KEGG or Biocartia or GO. The differentially expressed genes twofold up-regulated and down-regulated genes (*p* < 0.05) were further analyzed with Ingenuity pathways analysis (IGE). We classified functional groups, pathways and networks according to the importance the group of genes in each comparison. We highlighted functional groups, pathways and networks in each comparison based in relevance for gangliogenesis.^3^

For the PCA: Background correction, normalization, expression calculation, and PCA generation were performed by affystart, a package within affycoretools (MacDonald, 2019) run in R 3.5.2 by R Core Team (2018).

Heatmaps: Heat maps based on macroarray signal intensities for significant genes were generated in pheatmap 1.0.12 (Kolde and Kolde, 2015) run in R 3.5.2 by R Core Team (2018).

### Whole Mount *in situ* Hybridization and in Cell Cultures

Antisense, digoxigenin-labeled RNA probes are prepared from linearized templates. Whole mount *in situ* hybridizations are performed as described in https://media.bcm.edu/documents/2014/28/insituhybridization.pdf.

### *In situ* Hybridization on Paraffin Sections

For *in situ* hybridization on paraffin sections, embryos were fixed in modified Carnoy’s solution. After dehydration and paraffin sectioning at 8–10 μm, *in situ* hybridization was carried out as detailed in Etchevers et al. (2001), except that the slides are not treated with proteinase K.

### Immunohistochemistry

For post-in situ immunohistochemistry with HNK1, slides were blocked for 30 min in 5% donkey serum in PBS. Slides were incubated in 1:100 HNK1 or HuC/D (Sigma clone 15A7.1) for 4 h. Immunoreactivity was visualized a rhodamine red X conjugated mouse IgM (Jackson Immuno Research) at 1:300. See Key resource Table for antibody source (Supplementary Table S4).

### Injection of Cells Into the Chick Embryo

Embryos were staged according to the number of somites formed. A window was cut in the eggshell and a 1:25 mixture of India ink and Ringer’s solution injected into the sub-blastodermal cavity to reveal the embryo. DiI (cell tracker CM-DiI, C-7001, Invitrogen/Molecular Probes) was prepared by diluted the lyophilized contents of the vial in ethanol (1/10) in 10% sucrose. Vital labeling of U20S cells with DiI was as follows. Cells were trypsinized and resuspended in plain DMEM and incubated for 20 min at room temperature with DiI mix, then washed three times with ice cold DMEM (centrifuging resuspended cells at 1,000 rpm for 15 min). After the three washes, pellet cells were injected into various locations in the embryos.

Fertilized eggs were incubated at 38°C for ∼28 h, until embryos reached HH10-12 (for cranial NCC) and HH16 (for tNCC). Eggs were windowed and visualized by a sub-blastodermal injection of India ink (diluted 1:10 in PBS). A small amount of U2OS cells (10 μl) labeled with DiI were injected under cranial ectoderm by a pulse of air pump, or at trunk levels. The eggs were closed with Scotch tape and reincubated for an additional 24 h. Embryos were removed from the eggs, stripped of the membranes, and fixed in 4% paraformaldehyde overnight before being stored in PBS.

Wholemount immunostaining was as follows. Embryos were thoroughly washed in PBS, and then blocked overnight with PBS containing 1% Triton-X100 and 10% FBS at 4°C. After 3 h at room temperature in washing buffer (PBS with 1% Triton-X100, 1% FBS), embryos were incubated with 1:300 HNK1 supernatant in PBS overnight at 4°C. The next day, embryos were extensively washed and incubated with an anti-mouse IgM-specific Alexa 488 conjugated antibody (Invitrogen). The following day the embryos were washed extensively and Z-scanned with a 410 LSM confocal microscope under a 4x magnification and projected into a single file with LSM 5 Image Browser by Zeiss.

The U20S cells, human bone osteosarcoma epithelial cells (ATCC < *cps*:*sup* > ® < /*cps*:*sup* > HTB-96^TM^), and the cell-line U2OS-MIF secreting cells were kindly provided by Dr. Rimas Orentas, Seattle Children’s Hospital, Seattle. These cells were permanently transfected with MIF, thus they are a stable source of MIF.

### TNCC-Enriched Cultures From Trunk NT Explants

Cultures were prepared by following the techniques previously described in past research (Bronner-Fraser and Garcia-Castro, 2008; Walheim et al., 2012). Briefly, chicken eggs were incubated to HH13-17. Embryos were extracted from their yolk into autoclaved Ringer’s where extra-truncal embryonic tissue was removed. Remaining tissue was incubated at 37°C in 5% CO_2_ in DMEM-diluted Dispase [0.24 U/ml; Roche (Indianapolis, IN, United States) and Cell Systems (Kirkland, WA, United States)]. Embryos were generally incubated for 75–90 min then placed in L15 for isolation of the NT with fine tools to obtain clean NT. Isolated NTs were cut into segments 1–4 somites long and cultured overnight on a fibronectin-coated surface in (DMEM L-Glutamine 2 mM, and antibiotics (Penicillin G: 90–100 U/ml, Streptomycin: 90–100 μg/ml, all Omega Scientific; Tarzana, CA, United States), and 8–10% FBS (Omega Scientific or Life Technologies) to allow for tNCC emigration and halo formation around the NT. In general, cells within resultant tNCC-enriched cultures that exhibited mesenchymal morphology and were not part of the NT were considered tNCCs.

### NT Electroporation for MIF

NCC electroporation was performed following (De Bellard and Bronner-Fraser, 2005; De Bellard et al., 2018). Briefly, a plasmid mix of control pMES-GFP or experimental pMES-MIF (2.5 μg/μl) inside a capillary needle was injected into the NT of a HH15 chicken embryo with a mouth pipette. Embryos were cultured for 3 h post-electroporation (HPE) and NT isolated for culture as described in section TNCC-Enriched Cultures From Trunk NT Explants. The electroporator (made in-house at Cal Tech) was set to 18 V, 5 pulses (50 ms on; 100 ms off), with electrodes flanking the NT.

### Boyden Assays

Standard Boyden assay: tNCC isolation involved culturing HH15-16 NTs overnight, removing NTs, gently lifting tNCCs by replacing media with DMEM/20 mM EDTA for 30 min and then adding Trypsin (2 min). tNCCs were washed twice in DMEM and plated on the fibronectin-coated 8.0 μm pore inserts (120,000 cells/well) of Boyden chambers (BD, Franklin Lakes, NJ, United States) containing DMEM. Treatment molecules were added to the bottom compartment. After 5 h incubation, cells remaining on the filter’s top side were removed by scrubbing with a cotton swab. Cells on the bottom were fixed, stained with toluidine blue and counted. Other proteins were also evaluated at times using the same controls, but not described.

Boyden assays were repeated using the immortalized cell line SpL201 (Lobsiger et al., 2001). These cells were lifted from culture dishes with Trypsin, washed with plain DMEM and plated on 8.0 μm pore inserts (120,000 cells/well) of Boyden chambers. These cells did not need fibronectin for proper attachment (De Bellard et al., 2018).

### Real Time Quantitative PCR for the Verification of Differential Expressed Genes

QPCR primers were designed by Primer 3 online to generate short products (100–300 bp, Tm about 60°C) and were synthesized by OPEN Biosystem. The sequences of the primers are listed in Supplementary Table S3. For each gene, QPCR was performed with RNA from three individual sample of each stage. The first strand of cDNA were synthesized using Invitrogen superscript III cDNA synthesis kit. QPCR was performed in triplicate for each cDNA with SYBR green Supermix Universal kit (Invitrogen) in ABI7300 (Applied Biosystems, Foster City, CA, United States). Blank controls were reactions omitting the templates. The RNA content of samples was normalized to housekeeping gene GAPDH (glyceraldehyde-3-phosphate dehydrogenase) amplification. The threshold cycle (CT value) at which a significant increase in PCR product is first detected, was recorded for each sample for the target genes and GAPDH. Only the genes that have a melting curve temperature more than 75°C were defined as successful amplification. ΔCT = CT of gene of interest minus CT of GAPDH. The fold change for each comparison was calculated following standard protocols.

### Ibidi Chemotaxis Experiments

The procedure was performed as described before (Walheim et al., 2012). Briefly, isolated trunk neural tubes from HH16 chicken embryos were isolated by incubating trunk pieces in Dispase (0.24 U/ml; Roche, Indianapolis, IN, United States) for 30–60 min at room temperature. Neural tubes (NT) were placed in L15 for isolation of the NT with fine tools (at times with small amounts of non-NT tissue remaining attached). NTs were cut into segments 1–4 somites long and cultured overnight on a fibronectin-coated Ibidi chamber in DMEM, L-Glutamine (1.8–2 mM), and antibiotics [Penicillin G (90–100 U/ml), Streptomycin (90–100 μg/ml)] (all Omega Scientific; Tarzana, CA, United States), and 8–10% FBS (Life Technologies) to allow for tNCC emigration and halo formation around the NT. Next day cells were exposed to a focal source of MIF or NGF (200 ng/ml) and imaged in a Zeiss 35M at 90 s intervals for 3 h. The captured images were converted into an ImageJ movie and analyzed using the Ibidi plugin for tracking individual cells.

### Wound Assays

Isolated trunk neural tubes from HH16 chicken embryos were isolated by incubating trunk pieces in Dispase (0.24 U/ml; Roche, Indianapolis, IN, United States) for 30–60 min at room temperature. NT were placed in L15 for isolation of the NT with fine tools (at times with small amounts of non-NT tissue remaining attached). NTs were cut into segments 1–4 somites long and cultured overnight on a fibronectin-coated surface in DMEM, L-Glutamine (1.8–2 mM), and antibiotics [Penicillin G (90–100 U/ml), Streptomycin (90–100 μg/ml)] (all Omega Scientific; Tarzana, CA, United States), and 8–10% FBS (Life Technologies) to allow for tNCC emigration and halo formation around the NT. Next day a wound was performed by scrapping with a pipette tip. Culture media was replaced with feeding media or with media containing MIF or NGF (200 ng/ml); cells were allowed to migrate for 4hrs before fixing with 4% PFA and staining with toluidine blue. We counted at least in 6–7 areas the number of cells that invaded the wound. Each experiment had a duplicate and the data corresponded to 5 experiments. Experiments were performed in plain DMEM.

## Results

### Purification of Migratory and Post-migratory Neural Crest Cells

Our working hypothesis was that the cessation of migration should involve up-regulation of new genes that cause cell aggregation and/or prevent further migration as well as down-regulation of genes required for cell motility. In order to find such genes, we compared the gene expression profiles between post-migratory cells with actively migrating NCCs. For this comparison, we developed an approach for purifying populations of trunk NCC (tNCCs) at distinct phases of migration in order to enrich for migrating cells versus condensing NCC populations.

**FIGURE 1:**
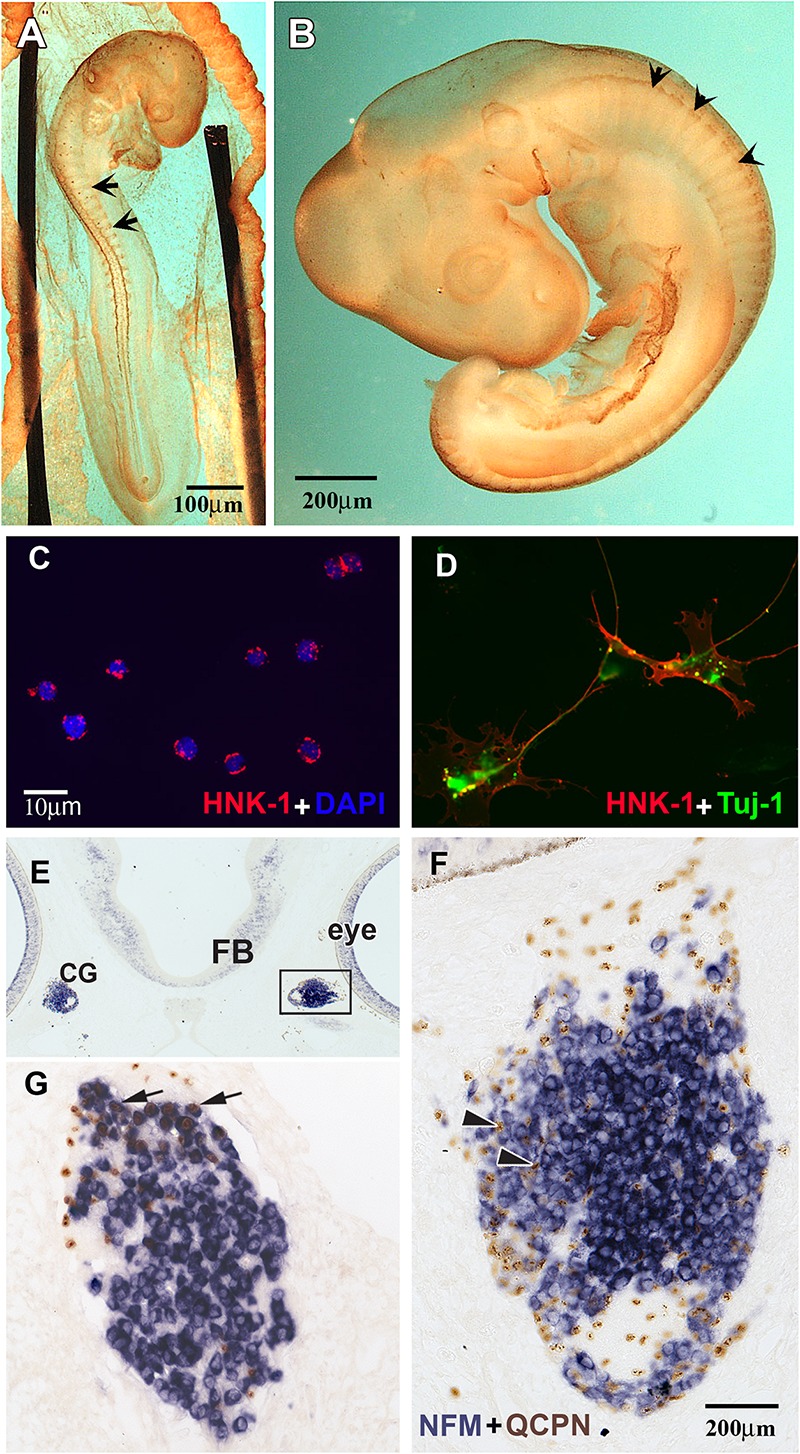
HNK1 identified migrating NCC and forming ganglia. **(A,B)** Whole HNK1 immunostaining (brown) on HH16 (E2.5, 26–28 somites) and HH19 (E3, 37–40 somites) chick embryos. **(A)** At HH16, tNCC (arrows) had begun migrating out of the neural tube. **(B)** By HH19, tNCC were beginning to coalesce into DRG (arrowheads). **(C,D)** Purified NCC were counterstained with DAPI to reveal all nuclei. HNK1 + cells (red) and DAPI + cells (blue) were counted to calculate the purity of the cell preparation. **(D)** When purified NCC were cultured overnight, they extended processes and expressed the neuronal marker, Tuj-1. **(E–G)** Purified quail (QCPN+, brown) NCC were injected into the head mesenchyme of 8–11 somites embryos and harvested 4 days after transplantation. **(E)** Grafted NCC were able to migrate to their normal targets such as the ciliary ganglia **(G)**, which are located adjacent to the eye. **(F)** Higher magnification of the boxed area in E showed that numerous donor cells could be found in the ciliary ganglion 4 days after operation. Most grafted cells incorporated into the ganglion, some of them were either undifferentiated or would become glial cells (arrowheads). **(G)** A section through the ciliary ganglion from another grafted embryo. Many quail NCC differentiated into NFM + neurons (arrows) while some are non-neuronal or undifferentiated cells.

We took advantage of the fact that chicken tNCCs express high levels on their cell surface of the antigen, HNK1, shortly after emigration from the neural tube all the way to the time of gangliogenesis (Strobl-Mazzulla and Bronner, 2012; Simoes-Costa et al., 2014; Giovannone et al., 2015). We started by isolating trunk regions from HH16 (Figure 1A), this stage is characterized by the robust level of migrating tNCCs (Giovannone et al., 2015). The other set of cells were isolated from the trunk of HH19 chicken embryos that corresponds to tNCCs that are beginning to condense into dorsal root ganglia (DRG) (Figure 1B). These two set of chicken trunks tissues were dissected free of the neural tube, notochord, dissociated into a single cell suspension and separated from mesenchymal cells by immune-selection.

To assess the purity of the isolated populations, the final cell eluate was subjected to cytospin right after the selection to determine the percentage of HNK1 positive cells. We consistently obtained 95–99% purity (Figures 1C,D) contrasting with the ∼5% HNK1 immunoreactive cells of the starting population. To further corroborate that these cells were true tNCC, we assayed the purified cells for Sox10 expression, and found that nearly all of the freshly isolated HNK1 + cells also were also Sox10 + (data not shown). However, it is important to corroborate that our isolated cells are capable of behaving like true tNCC. We next tested whether purified tNCCs from HH19 embryos were able to differentiate into normal NCC derivatives *in vitro* and *in vivo*. We found that our purified tNCCs were fully viable in culture and able to differentiate into neurofilament and Tuj1 positive neurons and extended neurites (Figure 1F).

In order to test the ability of our isolated tNCCs to be able to migrate and differentiate *in vivo*, we repeated the HNK1-positive cell isolation from quail embryos and grafted these tNCCs into chicken hosts at HH10. Quail donor cells can be distinguished from chicken host cells by using the quail specific antibody, QCPN (Le Lievre and Le Douarin, 1975; Rothman et al., 1990). After 3–4 days post injection, we found that QCPN+ quail cells contributed to NCC derived ganglia and were capable of differentiating into neurons (as assayed by HuC/D) (Figures 1E–G). Taken together, these results showed that our purified tNCC cells are viable, healthy, and able to migrate and differentiate normally into neurons after immune-selection. We did not test for glial differentiation in these transplants since Schwann cells do not appear until later in chicken, about embryonic day 6 (Rudel and Rohrer, 1992).

### Profile of Genes Up-Regulated in Late Migrating Neural Crest Cells

After confirming the purity and capabilities of our isolated tNCCs from both migrating (HH16) and condensing (HH19) phases of development, we carried out our goal of building a comparative hybridization of these two populations to screen for candidate genes up and down regulated at the end of migration. To this end, we generated and arrayed a cDNA library from embryos encompassing mid- to late-migrating NCC, and compared clones up or down regulated at HH19 versus HH16. Those with at least twofold or more difference in intensities were selected and 5′ sequence information was obtained. In total, we picked 995 clones that corresponded to 774 predicted cDNAs (Table 1). Isolated genes fall into diverse functional categories; we have provided a table of their distributions and assigned functions from Gene Ontology (Supplementary Table S1).

**TABLE 1:** Genes isolated from screen comparing HH16 with HH19 tNCC.

Categories	Unique genes	Total transcripts
Cell proliferation	18	59
Chromatin	14	53
Cytoskeleton	16	40
ECM	22	31
G protein/ras/rho	16	24
Signal molecules and receptors	16	33
Intracellular signaling	20	47
RNA binding	9	19
Transcription	17	69
Cell death	10	21
Nucleocytoplasmic transport	11	17
RNA processing	17	54
Protein prod. and degradation	26	86
Protein transport	21	49
Mitochondria/metabolism	7	60
Miscellaneous	8	8
Unknown/hypothetical protein	104	104
	352	774

One relevant observation was that many genes isolated were involved in chromatin remodeling (HMG family members and histone modifying proteins) as well as transcription regulators. The transcription regulators included tissue-specific factors such as PAX3, AP-2. Moreover, many RNA processing genes were enriched like PESCADILLO, WDR57, DEAD Box protein 54, and NONO. To further corroborate these findings, we selected some of the genes and performed *in situ* hybridization as a secondary screen to verify their expression in NCC derivatives.

#### Ligand/Receptor/Signaling Molecules Isolated From the Screen

**FIGURE 2:**
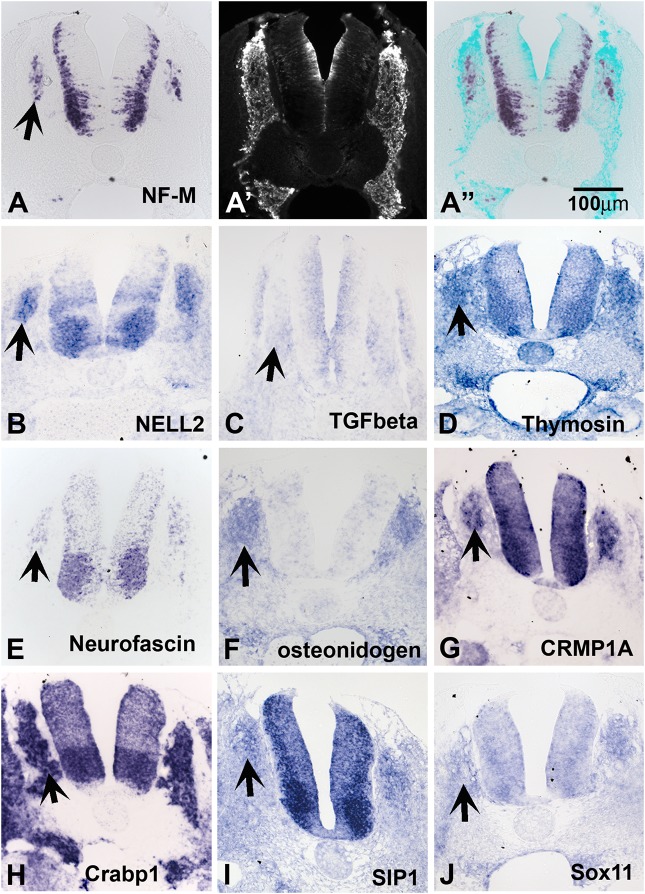
Expression of known genes. Chicken section *in situ* for set of genes. **(A)** NF-M. **(A’)** Shows section immunostained with HNK1. **(A”)** Shows overlap image of section for HNK1 and NF-M. **(B)** NELL2. **(C)** TGFbeta. **(D)** THYMOSIN. **(E)** NEUROFASCIN. **(F)** OSTEONIDOGEN. **(G)** COLLAPSIN response mediating protein 1A, CRMP1A. **(H)** cellular retinoic acid binding protein 1, Crabp1. **(I)** SMAD interacting protein 1, SIP1. **(J)** SOX11. Arrows point to DRGs.

Because late-migrating NCC are beginning to differentiate into their derivatives, we anticipated finding a large number of genes involved in neuronal differentiation. Accordingly, we found a good number of transcripts that encode neuronal markers; these include neurofilament M, Hu proteins, and ubiquitin c-terminal hydrolase. *In situ* hybridization on transverse trunk sections showed that these genes are expressed in the inner core of the dorsal root and sympathetic ganglia (SG) (Figure 2A for NF-M and others).

We found that many genes involved in neuronal signaling events were upregulated at the conclusion of tNCC migration (Supplementary Table S1 GO genes). Among the most prominent because of their known role during tNCC development are WNT11, FGFR1, VEGF receptor and Neuropilin 2.

Our screen showed NELL2 (neural EGF-like ligand 2), an extracellular ligand which has been shown to be expressed in the newly differentiated neurons in the DRG as well as neural tube (Nelson et al., 2004; Figure 2B). We found that the well-known signaling molecule TGFbeta (Wurdak et al., 2005) is expressed diffusely in the apical layer of the NT and a subpopulation of the DRG (Figure 2C). Thymosin beta 4, a gene linked to actin dynamics, was observed in condensing DRG and SG (Figure 2D). We isolated several members of the CRMP family (1A, 1B, 2B, and 4B). Their expression patterns nicely matched that of Neurofascin (Figures 2E,G), also isolated in our screen. CRMPs have been known to regulate actin dynamics as well as Semaphorin/Neuropilin signaling which affects both axonal pathfinding and NCC migration. A true novel finding was the presence of osteonidogen, this gene encodes a cell-ECM adhesion as part of the BM complex, a member of the Nidogen family of basement membrane proteins that binds collagen and are important for proper neuronal stability (Figure 2F; Wolfstetter et al., 2019).

#### Transcription Factors

Among our genes we isolated many transcription factors previously implicated in NCC development such as PAX3, AP-2 alpha, and SALL4. We also found robust expression in DRG and NT for a member of the acute phase response signal pathway, CRABP1, a member of the retinoic acid signal pathway, but its role in the NCC migration and differentiation is not clear (Figure 2H). In addition, we identified factors not previously known for their involvement in NCC/DRG development such as the homeobox transcription factor SMAD interacting protein 1 (SIP1, Figure 2I) and SOX11 (Figure 2J) and. The expression patterns of SIP1 and SOX11 revealed that they are both present in the developing DRG and SG.

#### Potentially Novel Genes Isolated

**FIGURE 3:**
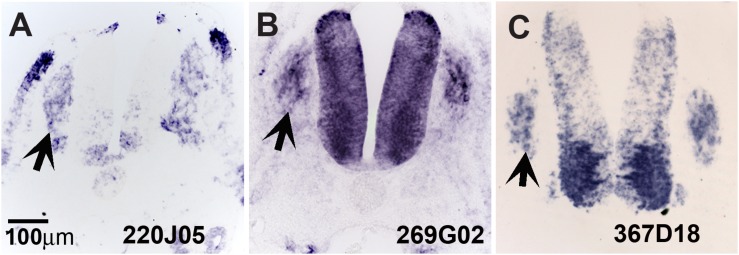
Expression of unknown genes. Chicken section *in situ* for set of 3 unknown genes. **(A)** 220J05. **(B)** 269G02. **(C)** 367D18. Arrows point to DRGs.

**FIGURE 4:**
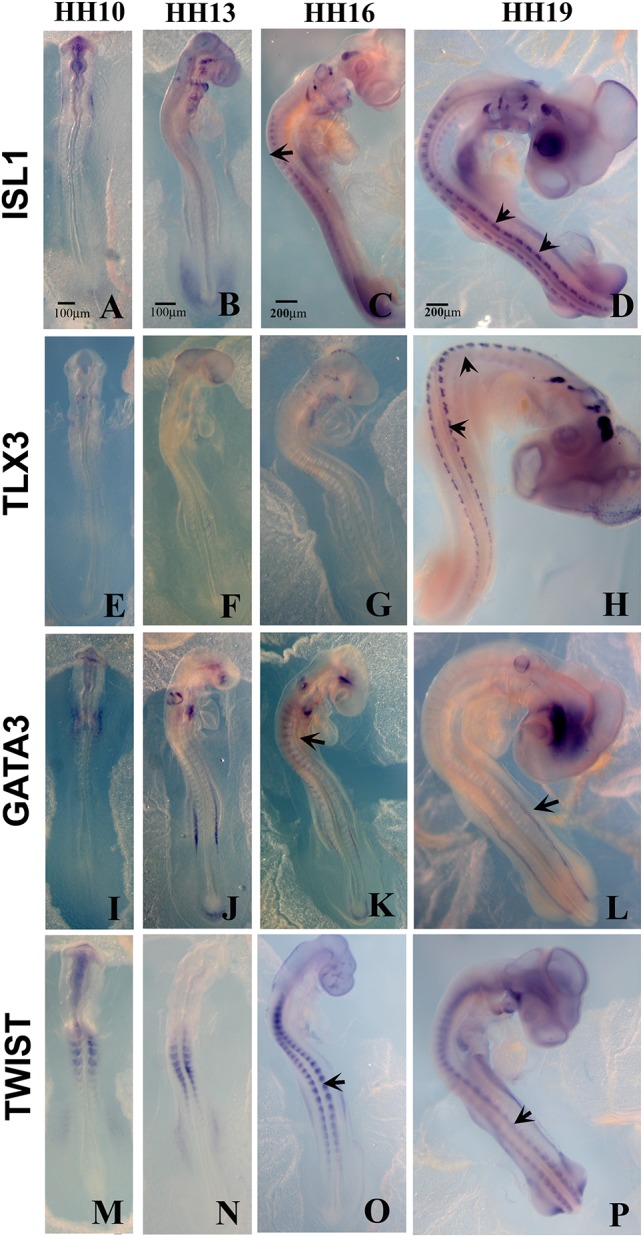
Expression of upregulated genes. Chicken wholemount *in situ* for set of 4 upregulated genes at HH10, HH13, HH16, and HH19 chicken embryos. **(A–D)** ISLT1. **(E–H)** TLX3. **(I–L)** GATA3. **(M–P)** TWIST. Arrows point to migrating tNCC or coalescing DRGs. Scale Bars are for microns.

Our screen showed 104 of the 774 clones (∼13%) with either (a) no homology to genes in the available databases, (b) on unassigned chromosomes, or (c) correspondence to ESTs of unknown function (Table 1). Many of these novel genes exhibit intriguing expression patterns in the NCC (Figure 3). Several of the unknowns are present in the inner core of the DRG as well as the select areas of the neural tube (Figures 3A–C). Importantly, many had partially overlapping but distinct patterns of expression. For example, clone 220J05 is expressed in the dermomyotome, DRG, SG (data not shown), ventral neural tube as well as the dorsal tip of the neural tube (Figure 4A), whereas 269G02 is expressed intensely in the neural tube (except for the most dorsal region) and the floor plate as well as DRG and SG (Figure 3B). In addition to DRG and SG, 367D18 is expressed in ventral and the marginal zone of the neural tube (Figure 3C).

### Functional Categorization of tNCC Genes Changed Between HH19 and HH16

In order to identify gene ontology categories, we used the DAVID functional annotation tool. The gene category enriched in HH19 tNCC compared to HH16 is shown in Supplementary Table S2. From this comparison we were able to identify many well-known genes known to be involved in NCC development as well as new ones (Supplementary Table S2). We then went on to verify our screen results by *in situ* hybridization and/or QPCR. Table 2 shows results from QPCR for a set of genes, showing that our findings closely follow those by QPCR comparison between HH16 and HH19 tNCC.

**TABLE 2:** Quantitative PCR verification of the HH19 up-and down-regulated genes compared with HH16 tNCC.

Gene symbol	Gene description	Q-PCR fold change	Macroarray fold change
TLX3	T-cell leukemia, homeobox 3	138.48	9.49
ADCYAP1	Adenylate cyclase activating polypeptide 1	24.88	13
MAB21L1	Mab-21-like 1 (*C. elegans*)	18.77	14.38
CFC1	Cripto, FRL-1, cryptic family 1	5.87	2.67
STMN2	Stathmin-like 2	7.22	6.87
SANAP25	Synaptosomal-associated protein, 25kDa	2.87	4.53
Similar to ADAM12	Similar to ADAM 12 (2.99)	5.11	2.99
TBX22	T-box 22	–9.52	–9.09
FGFR2	Fibroblast growth factor receptor 2	–4.90	–5.05
Osteopontin	Osteopontin	–12.04	–4.38
OSF2	Periostin, osteoblast specific factor	–6.94	–4.16
SEMA3C	Semaphorin 3C	–37.04	–11.39
FZD7	Frizzled homolog 7	2.26	3.26
CRABP1	Cellular retinoic acid binding protein 1	3.11	8.49
RARB	Retinoic acid receptor, beta	0.92	−2.56

#### Up-Regulated Genes in HH19 tNCC Compared to HH16

From the up-regulated group of genes, we chose 4 genes ISLET, TLX3, GATA3, and Twist to verify by whole mount *in situ* hybridization that they are expressed in migrating tNCC (Figure 4). The results showed the expression pattern of the four genes in the chicken embryo during DRG development was consistent and supportive of our screen. ISLET1 (and ISLET2) are motor neuron marker that were up-regulated in the HH19 tNCC array (Supplementary Table S2A). TLX3 (Hox11) was identified as a transcription factor and a proto-oncogene with an important role in T cell leukemia (Lu et al., 1992). TLX3 has been known to be expressed in NCC-derived DRG and SG ganglia (Logan et al., 1998). GATA3 transcription factor, expressed in developing DRG (Figure 4C), has already been shown to be part of NCC GRN (Simões-Costa and Bronner, 2015). Finally, TWIST, a well-known NCC player, whose repression leads to cell dispersion (Chen and Behringer, 1995).

#### Down-Regulated Genes in HH19 tNCC Compared to HH16

**FIGURE 5:**
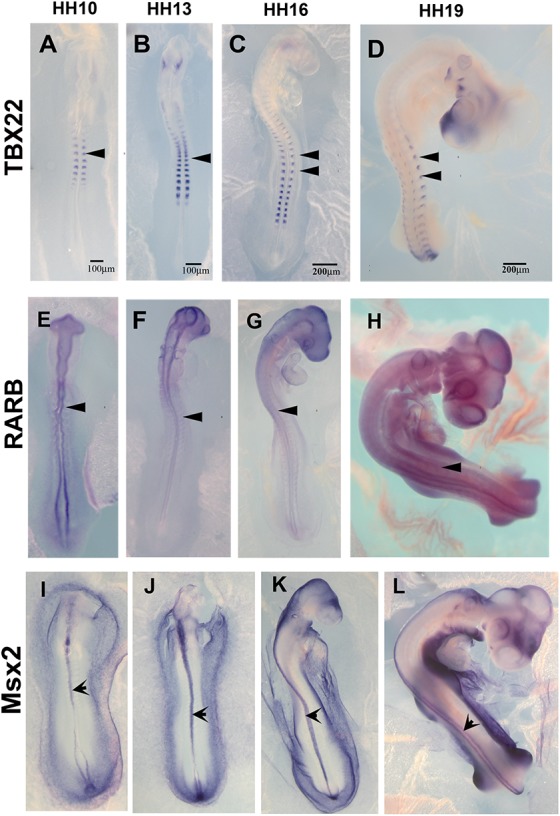
Expression of downregulated genes. Chicken wholemount *in situ* for set of 3 upregulated genes at HH10, HH13, HH16, and HH19 chicken embryos. **(A–D)** Tbx22, arrowheads point to migrating tNCC or coalescing DRGs. **(E–H)** RARB, arrowheads point to NT and faint expression in tNCC or coalescing DRGs. **(I–L)** MSX2, arrowheads point to NT. Scale Bars are for microns.

The list of down-regulated genes in Supplementary Table S2B shows 256 genes. Among these, we chose TBX22, RARB, and MSX2 to corroborate by *in situ* hybridization their expression in HH16 and reduction in HH19. We found that TBX22 genes were expressed in peripheral ganglia as shown before, and especially that its expression was high during tNCC migration (Figures 5A–D; Bush et al., 2002). This transcription factor is known to be important in cranial NCC-derived morphogenesis (Higashihori et al., 2010). RARB and MSX2, however, did not show distinguishable high expression in migrating tNCC, although expression was reduced by HH19 in our QPCR (Table 2). RAR-beta transcripts were present in a small subset of migrating NCC in the head of the HH10 embryo but down regulated in trunk DRG (Figures 5E–H). Finally, MSX2 is another well-known transcription factor that represses several NCC-derived cells (Takahashi et al., 2001; Gammill and Bronner-Fraser, 2003) that we found strongly expressed in NT and down regulated in HH19 NT (Figures 5I–L).

### Bioinformatics Analysis

**FIGURE 6:**
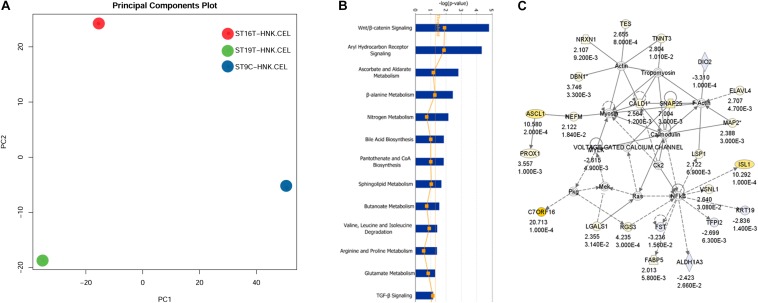
IGE for HH19 compared with HH16 macroarray. **(A)** Background correction, normalization, expression calculation, and PCA generation were performed by affystart, a package within affycoretools run in R 3.5.2 (R Core Team, 2018; MacDonald, 2018). **(B)** Focus pathways in HH19 versus HH16 differential expressed genes. **(C)** The top network in the HH19-HH16 differential expressed genes.

The first analysis we performed was a PCA analysis (Figure 6A). The main idea of PCA is to reduce the dimensions of a dataset that have a large number of interrelated variables, while the current variation in the dataset is maintained as much as possible. PCA methods transform the original variables into a set of linear combinations, the principal components (PC), which capture the data variability (You et al., 2013). PCA graph for HH9, HH16, and HH19 macroarray showed that each of our macroarrayed NCC population clustered apart, indicating that their mRNAs are quite distinct.

#### Ingenuity Pathway Analysis

Next, we carried out a set of analyses to determine ingenuity pathways. Using Ingenuity pathways analysis (IGE) we were able to identify that there were unique different groups of genes involved in different pathways between HH16 and HH19. The top network involved in the cellular movement, nervous system function and tissue morphology. In the network, the yellow color showed up-regulated genes and light blue showed down-regulated genes, the darker the color, the more significant change of the genes. Below each gene in the network is the fold change and *p*-values. The solid line indicates direct interaction between molecules and dashed lines indicate indirect interaction between molecules. Among the interesting pathways observed by IGE analysis was again the WNT/β-catenin pathway by HH16 tNCC (Figure 6 and Supplementary Table S2). This pathway was dominant among all the ones that IGE brought up. In this pathway, we observed upregulation of ASCL1, PROX1, NRXN1, TNNT3, ELAVL4, and ISL1 genes, while FST, TFPI1, DIO2, and ALDHA3 were down-regulated (Figure 6B). There are several functional groups of genes that were found to be involved in the development of DRG (see Supplementary Table S2, HH19-16 IGE groups). The largest group corresponded to differentiation of cells with 40 total genes, followed by 38 genes involved in cell movement, followed by nervous system development/neurogenesis with 18/14 genes. Thus, our macroarray was successful on the overall aim of finding key genes in DRG development.

The most important functional group we were able to distinguish with our analysis was the one involved in cellular development, nervous system development, cellular movement and organ morphology (Supplementary Table S1). There are 40 genes involved in the differentiation of the cells, 11 involved differentiation of neurons and 4 involved in the overall differentiation of ganglia. There are 18 genes related to the development of nervous system and 12 involved in the growth of neurites.

#### Heatmap forHH16 Versus HH19 in Comparison to HH9 (Cranial NCC)

**FIGURE 7:**
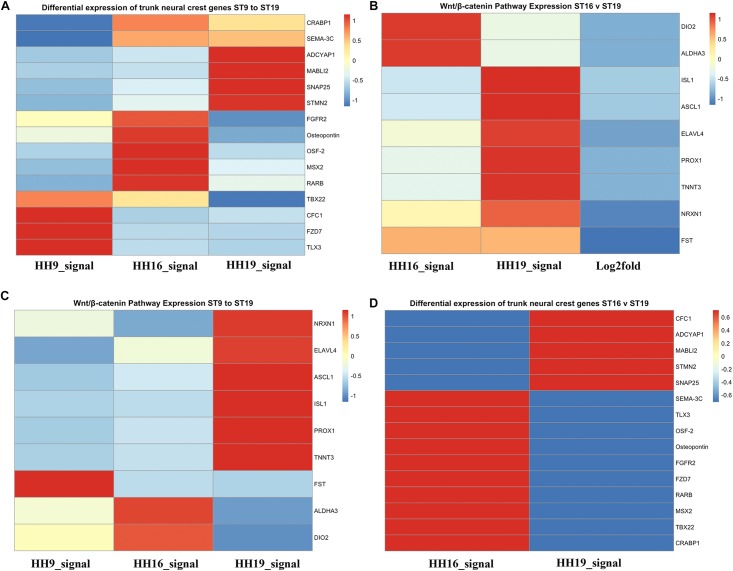
Heatmaps for HH9, HH16 and HH19 macroarray. **(A)** Heat maps based on macroarray signal intensities for significant genes were generated in pheatmap 1.0.12 run in R 3.5.2 (Kolde and Vilo, 2015; R Core Team, 2018). **(A)** Differential expression of trunk NCC genes HH9, HH16 and HH19. **(B)** WNT/β-catenin pathway expression of HH16 versus HH19. **(C)** WNT/b-catenin pathway expression of HH9, HH16, and HH19 comparison. **(D)** Differential expression of trunk NCC genes HH16 versus HH19. Heatmap color scale follows the sequential scales, blended progression, from the least (blue) to the most (red) shades, representing low to high values (blue to red scale, with white as almost no difference).

We also generated heatmaps for the main genes that gave the biggest differential expression across the three stages of development that we analyzed (HH9, HH16, and HH19, Figure 7). One of the first observations that stood out was that while some genes were highly expressed in migrating HH9 NCC they were reduced as development took place, for example TBX22, CFC1, FZD7, and TLX3 between HH9 and HH19 (Figure 7A). A similar pattern but in reverse was observed for ADCYAP1, MABL12, SNAP25, and STMN2 between HH9 and HH19 (Figure 7A). HH16 had FGFR2, Osteopontin, OSF-2, MSX2, and RARB higher than at HH9 and HH19 (Figure 7A).

One of the signaling pathways that stood out from our analysis was the WNT/β-catenin pathway. Heatmaps for this pathway showed clear distinct differences. While DIO2, ALDHA3 gene expression was high in HH16, but low in HH19 NCC; while in contrast ISL1, ASCL1, ELAVL4, PROX1, and TNNT3 were low in HH16 and high in HH19 (Figure 7B). On the other side, NRXN1, ELAVL4, ASCL1, ISL1, PROX1, and TNNT3 were low in HH16 but high in HH19 (Figure 7C). An overall comparison between HH16 and HH19 shows low levels CFC1, ADCYAP1, MABLI2, STMN2, and SNAP25 in HH16 and higher in HH19. Figure 7D shows contrasting levels of expression between HH16 and HH19 for SEMA3C, TLX3, OSF2, Osteopontin, FGFR2, FZD7, RARBB, MSX2, TBX2, and CRABPB1 expression (Figure 7D).

### Identification of MIF as a Putative Neural Crest Chemoattractant

Among the genes up regulated in post-migratory tNCC was the cytokine Macrophage migration inhibitory factor (MIF). While MIF was originally identified by its ability to inhibit random migration of leukocytes (Bucala, 1996), this cytokine shares signaling with Sdf1 (found in our screen) via the CXCR4 receptor, known to be involved in tNCC chemoattraction to the SG position (Kasemeier-Kulesa et al., 2010). Because of these interesting co-relations, we decided to test the role of MIF in tNCC migration.

**FIGURE 8:**
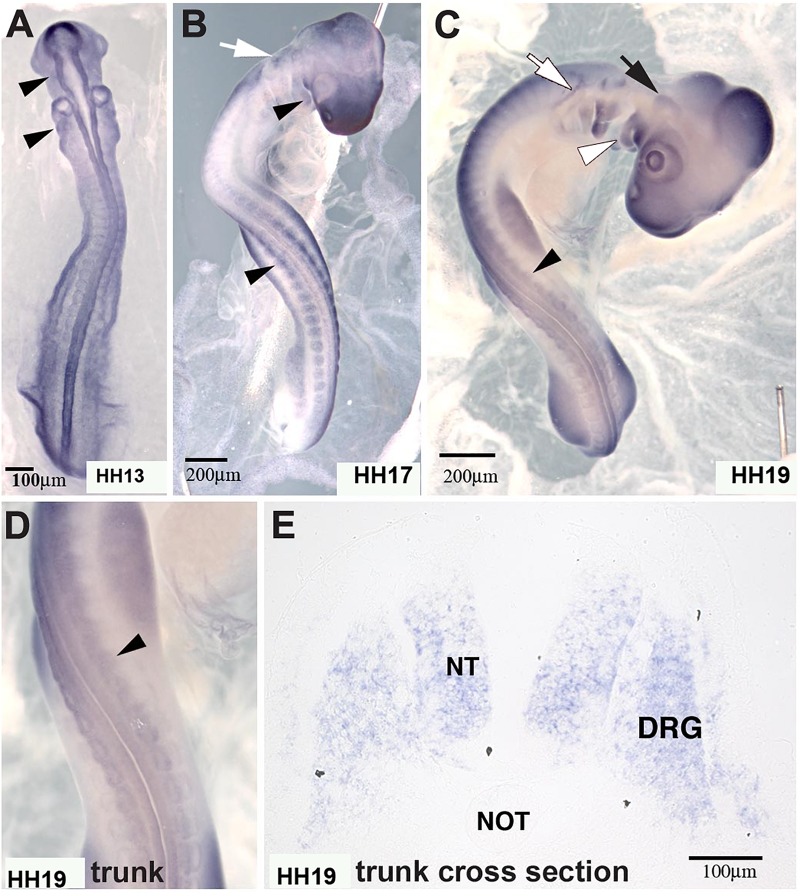
Macrophage migration inhibitory factor (MIF) was expressed by NCC. **(A)** MIF was expressed in migrating cranial NCC (arrowheads) at HH13 (St.13, E2, 16–20 somites). **(B)** By HH17 (St.17, E2.5, 29–31 somites), MIF transcript could be detected in branchial arches (white arrowhead), tNCC (black arrowhead) in addition to cranial NCC (white arrow). **(C)** At HH19 (St.19, E3, 37–40 somites), MIF was expressed in the trigeminal ganglion (black arrow), epibranchial ganglia (white arrow), branchial arches (white arrowhead) and DRG (black arrowhead). **(D)** Higher magnification of the trunk region in **(C)** showed MIF expression in the developing DRG (black arrowhead). **(E)** Section *in situ* hybridization performed with HH19 trunk transverse section demonstrated that MIF could be observed in the neural tube (NT) and dorsal root ganglia (DRG). Scale Bars are for microns.

*In situ* hybridization analysis of the distribution pattern revealed that MIF is expressed in the neural tube as well as in developing DRG and SG at the time that ganglia begin to condense (Figure 8). At earlier stages HH13 and HH17, it is also expressed in the somites through which tNCC migrate.

**FIGURE 9:**
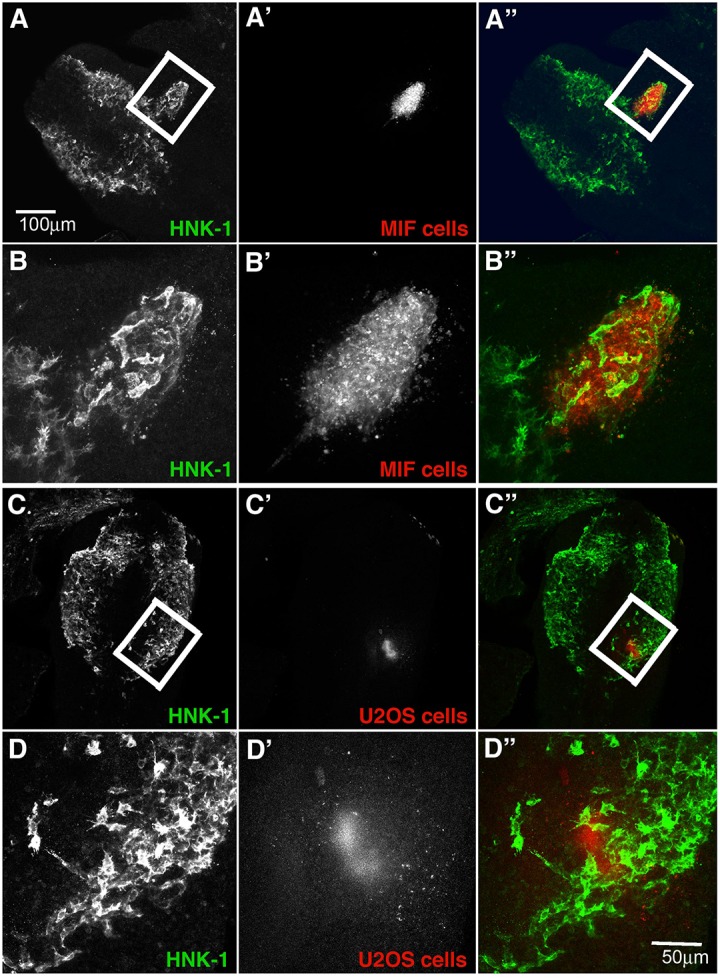
Exogenous MIF attracts tNCC *in vivo*. **(A,A’,A”)** Shows HNK1 (green) and, DiI U205 MIF-expressing cells (red), in the head of a HH10 embryo. **(B,B’,B”)** A higher magnification image taken from a confocal stack of the boxed area in **(A)**. Injected cells (red) were enveloped by HNK1 + NCC (green). **(C,C’,C”)** Shows HNK1 (green) and, DiI MIF cells (red), in the head of a HH10 embryo. **(D,D’,D”)** Is a higher magnification image taken from a confocal stack of the boxed area in **(C)**. Injected cells (red) NCC were not associated with the control cell clump (U205). Scale Bars are for microns.

The upregulation of this cytokine in forming ganglia raises the intriguing question of whether NCC cells may be providing autocrine signals. Although thought to be inhibitory for leukocytes, its expression in developing ganglia suggested that it may play and attractive rather than an inhibitory role for tNCC. To test whether MIF might serve as a chemoattractant *in vivo*, we injected MIF expressing cells outside of the normal NCC migratory pathways in live chicken embryos to see if NCC will be attracted to these cells and deviate from their pathway. Conversely, we tested its potential ability to repel NCC by placing MIF-expressing cells directly into a NCC migratory stream. Results show that NCC deviate from their normal pathways of migration and move next to MIF-secreting cells. When MIF cells are placed in an ectopic location in the head lateral to the normal pathways of NCC migration, we observed NCC mixing with DiI-labeled MIF cells (Figures 9A,B, *N* = 6). This suggests that NCC are preferentially attracted at a distance to MIF-secreting cells. In contrast, no effects were observed with control cells (*N* = 6, Figures 9C,D). Experiments were performed at both cranial and trunk levels with similar outcome (data not shown). Sections through one of these embryos showed that cranial NCC mixed extensively with MIF-secreting cells and not with control U20S cells (Supplementary Figure S1).

**FIGURE 10:**
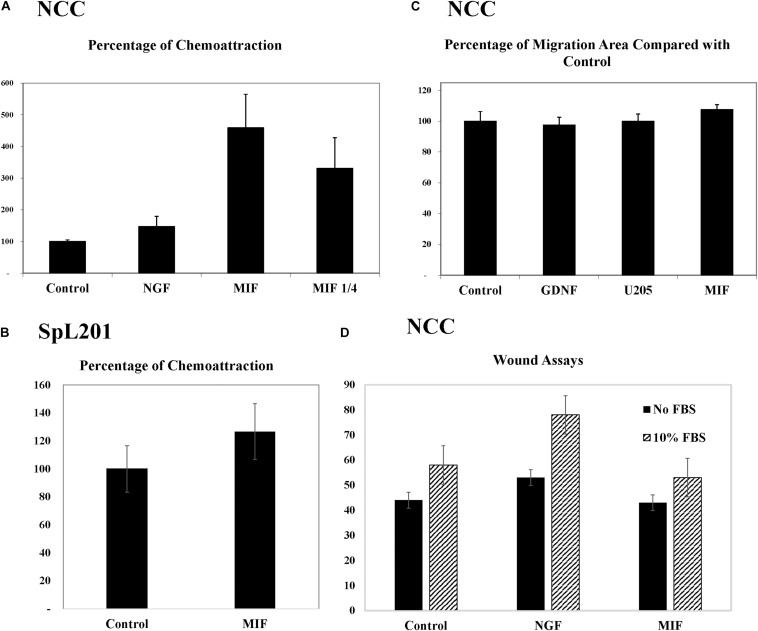
MIF attracts tNCC *in vitro*. **(A)** Isolated tNCC were seeded on top of Boyden chambers in the presence or absence of MIF and NGF. Number of cells were counted after 5 h of incubation. Data has been normalized to control numbers of tNCC in control wells (no chemokine present in bottom well. There were ∼4.5 times more tNCC in MIF wells compared with control or NGF. Diluting MIF to 1/4 decreased attraction by 35%. **(B)** Spl201 cells were exposed to MIF in bottom wells as performed for tNCC. **(C)** Isolated tNCC cultures were exposed to control media (control), U205 conditioned media or U205-MIF conditioned media overnight. The maximum migrated distance was measured. Graph shows results from 10 experiments combined and normalized to control media. **(D)** Isolated tNCC cultures were wounded and exposed to control media (control), NGF or U205-MIF conditioned media in the presence or absence of FBS in the media. Data in graph corresponds to number of cells in the wounds after 2 h of incubation. There was no statistical difference between all three treatments compared across. Only the presence of FBS increased wound healing in tNCC cultures, but there was no difference between control and MIF cultures.

To further corroborate these chemoattractive responses by NCC we examined the ability of MIF to stimulate chemokinesis and/or chemotaxis of tNCC cells in cultures. The first assay we performed was the Boyden chamber. Here, isolated trunk NCC were seeded on the top chamber in the presence or absence of MIF. After 5 h top cells were removed and those at the bottom counted. Results showed that MIF is a potent chemokinetic molecule, with trunk NCC migrating toward it 5X more compared with negative control or NGF as positive control (Figure 10A). We even observed a reduction in chemokinesis in the chambers when MIF was diluted 4 times. These results were replicated with an immortalized NCC cells line, SpL201, showing a 25% chemoattraction/chemokinesis by MIF (*p* < 0.03, *N* = 12) (Figure 10B).

Because Boyden assays indicate mostly chemokinesis rather than true chemotaxis (Muinonen-Martin et al., 2010), we looked at enhancement of migration of tNCC by two approaches. The first was to measure the maximum distance that tNCC migrated away from the NT, an indication that cells were moving faster if farther away. In this experiment we did not observe any difference between control, control cell line (U20S) and MIF-secreting cells (Figure 10C). The second was to do wound assays on tNCC halo. After 5 h we did not observe any difference between control or MIF treated trunk NCC, either in the presence or absence of FBS (Figure 10D), while we observed an increased wound healing in the presence of NGF.

**FIGURE 11:**
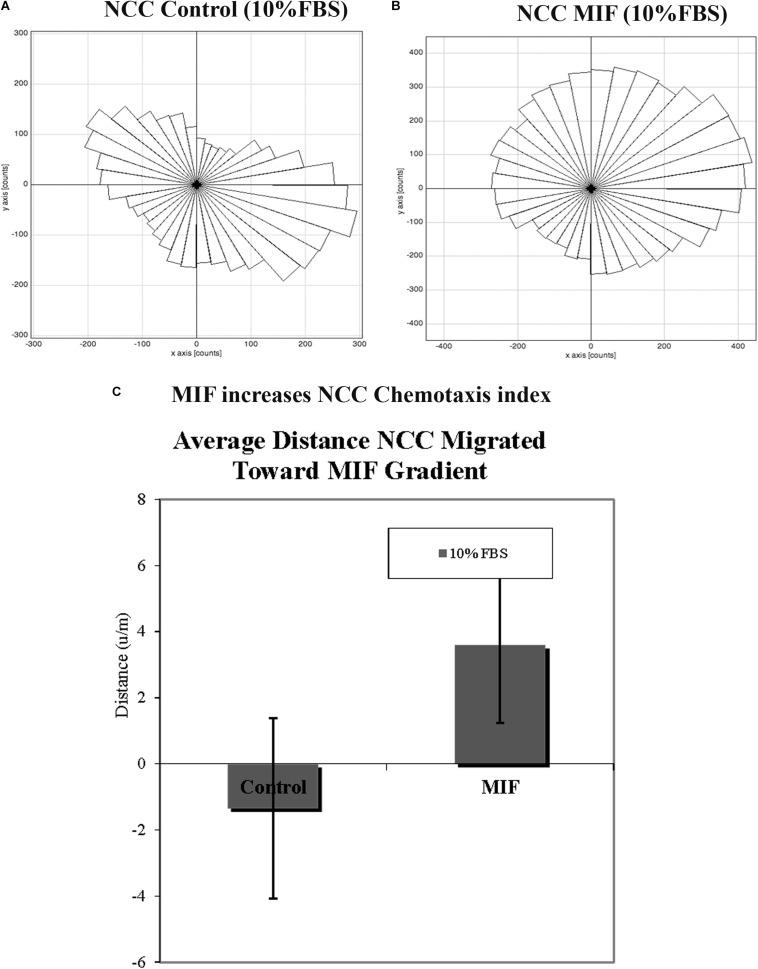
MIF attracts tNCC *in vitro*. Isolated tNCC were seeded Ibidi chambers in the presence **(B)** or absence of MIF **(A)**. Graph shows a rosette presentation of all the tracked paths. MIF was in the top chamber were we observed most of the tNCC in contrast to control that there are equal numbers of tracks in top and bottom portion of the graph. **(C)** Graph of chemotaxis index from **(A,B)** experiments. MIF index is significantly positive (attractant) compared with control media.

In order to corroborate MIF chemoattractant effect on tNCC we isolated trunk NCC as for Boyden chambers but plated them into IBIDI^C^ chemotaxis chambers. Cells were live imaged and tracked to determine their pathways. Results show that tNCC did not show preference in migration in the absence of MIF, but when MIF was added to one of the chambers, trunk NCC significantly migrated toward MIF (*p* < 0.03) (Figures 11A–C). All these findings were again further corroborated with the SpL201 cell line: these cells were strongly attracted to MIF in the presence of 2.5% FBS, and to a non-significant extent in 10%FBS (Supplementary Figure S2).

**FIGURE 12:**
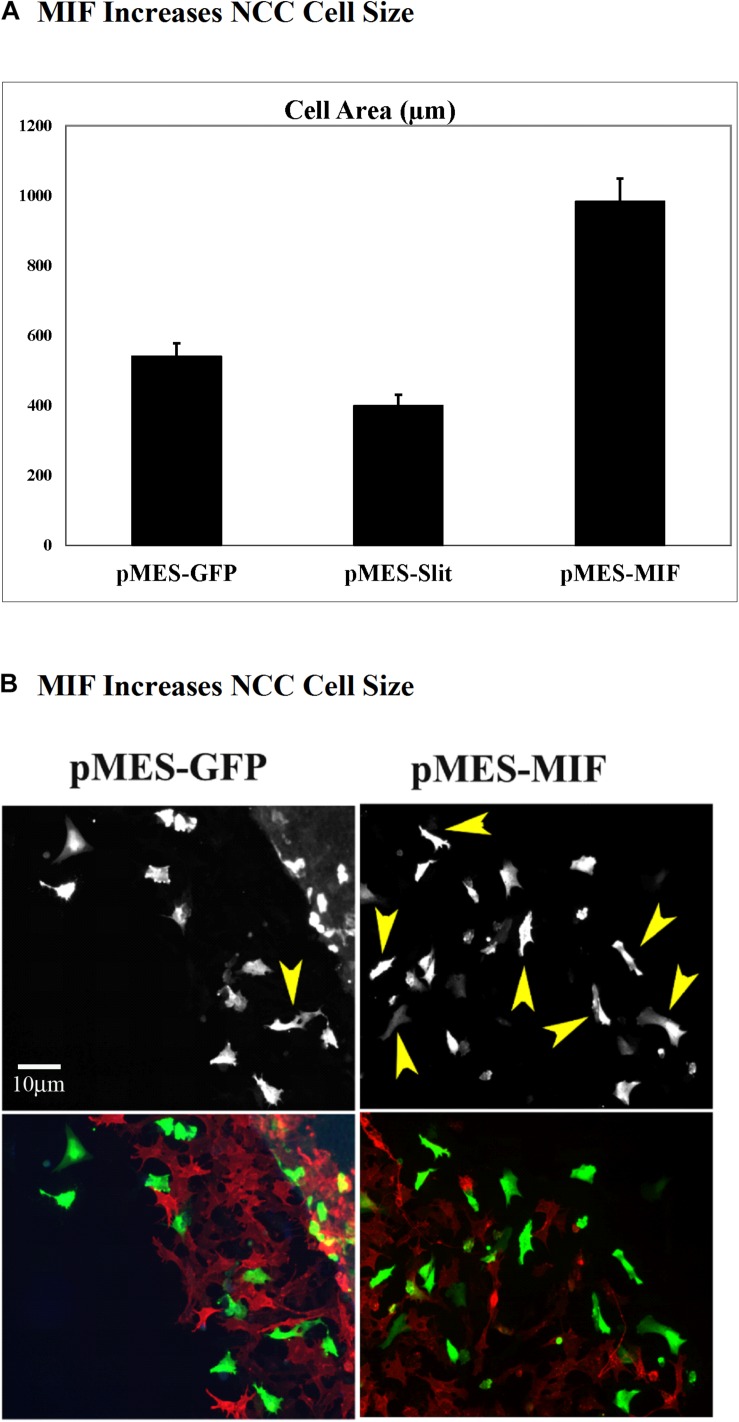
Expression of MIF by tNCC changes cell morphology. Chicken embryos NT were electroporated with pMES-GFP, pMES-Slit2, or pMES-MIF plasmids and cultured overnight. Next day cultures were fixed and tNCC cell areas measured (2D). **(A)** Graph for results from measuring more than 100 cells from at least 5 cultures. MIF doubled the cell area of tNCC compared with control, while Slit2 reduced their sizes. **(B)** Fluorescent images from one sample tNCC culture. Top panel corresponds to GFP channel for either GFP control or MIF-GFP. Bottom panel shows tNCC cultures overlap immunofluorescence with HNK1 (red) and GFP (green) plasmid expression.

Because migrating tNCC express MIF, and these cells respond with robust chemoattraction to MIF, we electroporated trunk NT in HH17 chicken embryos and cultured them to determine if tNCC expressing constitutively MIF affected tNCC mesenchymal morphology as it affected its migration responses (i.e., cell shape). We observed that indeed, tNCC MIF-expressing cells 2D area was larger than control or Slit2-expressing cells (Figures 12A,B; Giovannone et al., 2012). Further showing that MIF expression by tNCC is capable of changing cells behavior and the cell morphology from its classic mesenchymal shape.

## Discussion

The process of NCC delamination, migration, and final coalescing into peripheral ganglia is fundamental during embryogenesis. DRG formation is a completely different process from melanocyte migration or adrenal gland development since in gangliogenesis we have a modified MET mechanism, while this is not the case during melanocytes or chromaffin cell precursor migration. This study is the first of its kind as it searches for the molecules involved in the formation of DRG via subtractive macroarray of RNAs expressed in tNCC as they stop along their ventromedial migration and began to coalesce into what would eventually become a DRG (HH16 versus HH19 tNCC). Our preliminary findings show that: (1) a set of known and unknown genes are correlated with gangliogenesis, and (2) that MIF is a potent chemoattractant for tNCC. The results from this study provide new valuable information in our understanding of the process of DRG development and formation.

### Little Is Known About the Cessation of NCC Migration

NCC migration is a unique process given that these cells are true multipotent stem cells, have a wide range of target destinations and migrate long distances in the embryo (Le Douarin and Dupin, 2018; Tang et al., 2019). Along with their rapid migration throughout the embryo some of these migrating cells begin a unique transformation: they stop moving and start to aggregate into peripheral ganglia, condense to form cartilage, or incorporate themselves into other organs (Teillet et al., 1987). Recent studies on the NCC have greatly contributed to our understanding of its origin, fate, and migration but focus on the process of tNCC cessation of migration has been sparse (Duband et al., 1985; Teillet et al., 1987; Saito et al., 2012). Because of the paucity of information on the end stage of trunk neural crest migration, we took a comprehensive gene expression approach to identify potential genes involved in cessation of migration of tNCCs and its relation to gangliogenesis by looking at HH16 versus HH19 tNCC transcriptome with a macroarray.

Our transcriptome analysis is not the first to be performed with tNCC. The Bronner lab have recently shown the transcriptome for migrating cranial and trunk NCC (Simoes-Costa et al., 2014; Simoes-Costa and Bronner, 2016). These studies had brought great insights into the process of cranial NCC differentiation into its wide derivatives. Thanks to these studies we now know that it begins with the activation in subpopulations of migrating cranial NCC of unique GRN circuits which then lead to the turning on of lineage-specific gene batteries (Simões-Costa and Bronner, 2015). From these studies they were able to build a GRN for the induction and migration of cranial NCC (Simões-Costa and Bronner, 2015; Rothstein et al., 2018). However, these studies have not focused on the trunk NCC as this study does. One strength of our study is the novelty and uniqueness of our focus: a tNCC comparison between the migrating and beginning to coalesce tNCC. One weakness of our and others as well is that these isolated/selected NCC cells are a mixture of sensory-fated, autonomic fated and peripheral glia-fated NCC cells, and perhaps some melanocyte-fated cells. This “lumping” may mean that some lineage-restricted gene expression changes would be blurred, which would explain the wide range of transcription factors found: neuronal and other fates as well. Another and key one, is that positive gene expression is not equated with positive protein expression. Thus, our macroarray provides valuable information; that does not necessarily translate into quantifiable co-relation.

Comparing our screen and these two transcriptomes from the Bronner lab, we found only a few similar genes: FABP, MSX2, and EBF1 (Simões-Costa and Bronner, 2015). These very few similarities likely stem from the approach in both studies. Ours is a macroarray transcriptome built by comparing migrating HH16 tNCC with coalescing HH19 tNCC in DRG regions, while the other was by isolating the RNAs from migrating tNCC from HH14 embryo. Thus, both transcriptomes shed important light and likely complement each other in their results: Bronner’s shows RNAs from HH14 migrating tNCC while ours shows the RNAs differentially expressed in condensing tNCC, bringing up a new light into our understanding of the process of tNCC migration and formation of DRG.

Recently, Williams et al. (2019) have shown a global NCC GRN profiling of genes involved in their development. Our findings of upregulated/downregulated genes coincide in larger proportion with their migratory gene profiling. Further support that our array was focused of DRG transition is that we did not observe TFAP2B, Snai2, Zeb2, and Lmo4 (bona fide NC and EMT factors) in our array (Williams et al., 2019).

### Novel Candidates and Pathways

Of the 774 genes that we isolated from our screen, roughly 10–15% are unknowns or have no known function. These candidate genes may reveal new pathways in regulating NCC/DRG transitions and development. Our macroarray brought up the relevance that WNT/β-catenin pathway holds for NCC development, what was newer to some extent is its relevance in the crucial step of tNCC/DRG transition. Heatmap analysis of this pathway showed opposing expression levels of gene expression between HH16 and HH19. Most of the known role of this pathway relates with the cell differentiation of NCC as recently shown by Garcia-Castro’s lab (Leung et al., 2016). But also it has been shown to have a role in NCC migration *per se* (Sinnberg et al., 2018). Thus, it seems that WNT/β-catenin is the most wide range pathway in NCC development.

These novel genes may reveal new pathways in regulating NCC/DRG transitions and development in the future. The expression patterns of a subset of these unknown genes have specific, non-overlapping expression in NCCs and their derivatives. For example, 220J05, 269G02, and 367D18 have very strong expression in developing DRG (see Figure 3). We also uncovered interesting pattern in segregated expression for a set of known genes. NF-M, NELL2 (expressed on the medial DRG) and TGFbeta (expressed on the medial DRG) are observed in few cells, of the forming DRG. NELL2 (Neural EGFL Like 2), is expressed by DRG progenitors (Gurok et al., 2004) and is capable of promoting cell aggregation (Kim et al., 2014). These differences may be due to different NCCs populations as observed by Kulesa and co-workers, as between leader NCCs and trailing NCCs (Kulesa and McLennan, 2015; Dyson et al., 2018). Another possibility is that NELL2 and TGFbeta expression in central cells of the DRG is an indicator of beginning of neuronal differentiation. It is known that central DRG cells differentiate as neurons earlier than the peripheral DRG cells, and also that the first and largest neurons to differentiate are ventro-lateral and the dorsal smaller neurons follow later (Barber and Vaughn, 1986; Mu et al., 1993). Results from Confetti mice and chicken retroviral studies show that all migrating tNCC can give rise to its derivatives (Baggiolini et al., 2015; Tang et al., 2019). Thus, our observation of segregated populations within DRGs suggest that once tNCC settle into what will become the DRG, they begin quickly to differentiate into different cells. We would like to hypothesize that our *in situ* results uncovers the beginning of this tNCC differentiating into different set of cells within the DRG. DRGs are known for having its different neuronal types segregated into separate areas (Farinas et al., 1998; Liu et al., 2008; Chiu et al., 2014).

An interesting new finding was the finding of SIP1 in our array. Recently, Rogers and Newgreen labs showed that there are two distinct phases in the process of NCC EMT: first pre-migratory NCC lose their epithelial integrity, and then they can initiate mesenchymal transformation (Rogers et al., 2013; Simkin et al., 2014). First, detachment and then transformation into a mesenchymal cell, with this latter involving a novel requirement for SIP1 in order to regulate, in cranial regions, proper and timely cadherin expression during EMT completion in the cranial region. Thus, in an interesting EMT reversal, SIP1 is possibly involved in the MGT process that regulates NCC transition into non-mesenchymal phenotype.

Another important upregulated transcription factor was Phox2b, known to be expressed in NCC population that arises from the vagal level of the neural axis and that populates the stomach, midgut, and hindgut (Young et al., 1998). The homeobox gene PHOX2B is essential for the development of autonomic NCC derivatives (Pattyn et al., 1999). But of more relevance is that PHOX2B is important to promote, directly or indirectly, sympathetic neuron generation and control the expression of a large number of characteristic genes for sympathetic neurons differentiation (Stanke et al., 1999). Thus, PHOX2B may be regulating the expression of genes required for DRG neuronal differentiation. Interestingly, our findings of the specifier genes PHOX2B, GATA3, and ASCL1 in our array coincide with findings that these genes are involved in SG development (Morikawa et al., 2009). This coincidence suggests that SG and DRG may use similar regulatory genes during their differentiation.

Two upregulated genes that are “new and old” to NCC development were ASCL1 and Sema3D. ASCL1 is a pro-neural transcription factor (also known as MASH1 in mouse and CASH1 in chick), a crucial regulator of multiple aspects of neurogenesis in the central and peripheral nervous systems including progenitor cell maintenance, neuronal differentiation, and neurite outgrowth (Lo et al., 1991; Castro et al., 2011; Park et al., 2017). While ASCL1 role in neuronal differentiation is well-known, this is the first time that it is shown to be involved in the transition of tNCC from migrating cells to beginning of gangliogenesis by its presence in our screen.

SEMA3D is a member of the Semaphorins ligands which comprise one of the largest conserved families of axon guidance factors (Jongbloets and Pasterkamp, 2014). SEMA3F is expressed in the cranial NCC, dorsal neural tube and some migratory NCC (Gammill et al., 2006). SEMA3F lies downstream of WNT/TCF signaling in the molecular pathway thought to control cell cycle in NCC precursors. SEMA3F promoted fasciculation and modulates axon-axon interactions by regulating an adhesion molecule (Cloutier et al., 2004). Our macroarray data showing that SEMA3D is upregulated in late tNCC development brings a new function for Semaphorins: an autocrine role in developing DRGs.

Finally, results from IGE pathways suggest that NFkB, Calmodulin and cytoskeletal regulators are key pathways in HH16 to HH19 transition. NFkB is one of the best known signaling protein, and others have demonstrated that its pathway is involved in neurogenesis (Zhang and Hu, 2012), and gliogenesis (Fujita et al., 2011). Our study showing correlation between NFkB signaling pathways and DRG formation adds a new side to their story. Future studies will show if NFkB is involved in DRG neurogenesis and/or satellite glial cell formation. Calcium/Calmodulin-dependent protein kinase (or Calmodulin/BMP4) signaling pathways are known for a wide variety of effects during development (Massagué and Chen, 2000), probably the most striking is its role in cranial NCC beak formation (Jheon and Schneider, 2009). Our observation that this signaling pathway is also involved in DRG development adds a new twist Calmodulin/BMP4 wide repertoire of functions. Thirdly, while cytoskeletal regulators and changes are presupposed to be involved in gangliogenesis as required for neuritogenesis (McBeath et al., 2004; Neuhuber et al., 2004; Menon and Gupton, 2016), this is the first time that they are shown within this overall process of DRG formation.

### Macrophage Migration Inhibitory Factor (MIF) and Neural Crest Development

Macrophage MIF has been defined as a pro-inflammatory cytokine because it plays an important role in mediating immune responses in inflammatory processes (Bucala, 1996). Although, MIF can inhibit random migration of leukocytes, our findings show a complete opposite role for NCC: chemoattraction. Our results are in agreement with a recent study showing that MIF directs mesenchymal stem cell migration and infiltration toward tumor cells (Lourenco et al., 2015). Moreover, recently CXCR4 signaling has come up as critical in the cranial NCC transcriptome (Simoes-Costa et al., 2014). MIF is no stranger to tNCC derived cells, Nishihira and co-workers reported MIF is upregulated in Schwann cells during regeneration, and blocking MIF results in a reduced rate of cell regeneration (Nishio et al., 1999, 2002). Our *in vivo* and *in vitro* results demonstrate a new role for MIF: a potent chemoattractant for tNCC with no effect in chemokinesis (De Bellard et al., 2018).

There are no studies assessing the regulative role of MIF in NCC development. However, we know that the other CXCR4 ligand SDF1 is a chemoattractant and required for the establishment of tNCC in future SG next to the aorta (Kasemeier-Kulesa et al., 2010). Probably the most relevant study highlighting the role of MIF or Sdf1 in gangliogenesis is from the lab of Richard Miller showing that mice null for CXCR4 have abnormal DRG gangliogenesis (Belmadani et al., 2005). In addition, CXCR4 is involved in facilitating DRG clustering (Terheyden-Keighley et al., 2018). Thus, CXCR4 plays crucial roles in DRG development. Our study is the first to demonstrate that MIF may regulate the process that leads migrating tNCC to coalesce into DRG. One hypothesis we would like to present is that expression of MIF in the migrating tNCC might favor DRG ganglionation via self-attraction. MIF is highly over-expressed in many types of malignant tumors and its level of upregulation has been correlated with the aggressiveness of the cancers (Chesney and Mitchell, 2015; Liu et al., 2016). Interestingly, neuroblastomas, which are of NCC origin, express high level of MIF (Zhou et al., 2008). Thus, one of the ways that MIF affects DRG formation is by autocrine secretion, attracting the tNCC that expresses high levels of CXCR4 toward a coalescing DRG.

When found that the shape of tNCC changed and overall size cell growth occurred after MIF gain of expression. These events suggest that MIF might be one of the factors initiating the process that will lead a sub-population of migrating tNCC to coalesce together and begin DRG formation. Data from Vujicic et al. (2018) supports of this hypothesis, as they report that absence of MIF in mice make intestinal barriers more leaky and permeable to bacteria, thus absence of MIF leads to less tight adhesion between cells, a pre-requisite for migration. However, it is also possible that the increase in cell area *in vitro* is an indicator of increased cell-substrate adhesivity. Because we did not test the adhesivity of tNCC in our cultures, we cannot disregard this alternative explanation to cell shape change after MIF expression.

In summary, our findings show for the first time a set of genes involved in the cessation of tNCC migration during DRG formation. We also show that one of the genes, MIF, is a potent chemoattractant for tNCC.

## Data Availability Statement

The datasets generated in this study are publicly available in the Gene Expression Omnibus (accession: GSE139859).

## Ethics Statement

This study was carried out in accordance with the principles of the Basel Declaration and recommendations of United States Department of Agriculture (USDA) and the Department of Health and Human Services (DHHS). The animal protocol was approved by the CSUN Institutional Animal Care and Use Committee (IACUC).

## Author Contributions

VL design and did the macroarray, cell injection, and wrote the manuscript. SH and BG did the Boyden assays with Spl201 cells. JH supervised BG and wrote the manuscript. CC did bioinformatic analysis. MB did the Boyden assays with NCC, oversaw the *in vitro* studies by the students and wrote the manuscript.

## Conflict of Interest

VL was employed by the company Universal Cells Inc. at the time of this submission. The remaining authors declare that the research was conducted in the absence of any commercial or financial relationships that could be construed as a potential conflict of interest.

## Footnotes

1 http://www.biocarta.com/genes/index.asp

2 https://david.ncifcrf.gov/summary.jsp

3 https://analysis.ingenuity.com/pa/login/applet.jsp

## Supplementary Material

FIGURE S1 Exogenous MIF attracts tNCC *in vivo*. **(A–D)** Sections in an injected embryo shows HNK1 (green) and, DiI U205 MIF-expressing cells (red), in the head of a HH10 embryo. **(E–H)** A higher magnification image in **(A–D)** showing cranial NCC enveloping MIF expressing U205 cell. DAPI labels nuclei in blue. Scale Bars are for microns.

FIGURE S2 MIF attracts Spl201 *in vitro*. The NCC cell line Spl201 was seeded into Ibidi chambers in the presence **(B,D)** or absence of MIF **(A,C)**. Graph shows a rosette presentation of all the tracked paths. MIF was in the top chamber were we observe most of the Spl201 cells migrated in contrast to control that there are equal numbers of tracks in top and bottom portion of the graph. **(E)** Graph of chemotaxis index from **(A–D)** experiments. MIF index is significantly positive (attractant) compared with control media. **(F)** Migrated distance of Spl201 in presence or absence of MIF in the media.

TABLE S1 GO genes from HH16/HH19 macroarray comparison.

TABLE S2 Genes from HH16/HH19 macroarray comparison. **(A)** HH19-HH16 UP: up-regulated genes between HH16 and HH19 (433). **(B)** HH19-HH16 DOWN: down-regulated genes between HH16 and HH19 (256). HH19-16 IGE: Description HH19 to HH16 ingenuity pathway functional group-cellular movement. HH19-16 IGE groups: HH19-HH16 genes arranged by functionality.

TABLE S3 Q-PCR primers.

TABLE S4 Key resources table.

## References

[B1] Achilleos A. Trainor P. A. (2012). Neural crest stem cells: discovery, properties and potential for therapy. *Cell Res.* 22 288–304. 10.1038/cr.2012.11 22231630 PMC3271580

[B2] Acloque H. Adams M. S. Fishwick K. Bronner-Fraser M. Nieto M. A. (2009). Epithelial-mesenchymal transitions: the importance of changing cell state in development and disease. *J. Clin. Invest.* 119 1438–1449. 10.1172/JCI38019 19487820 PMC2689100

[B3] Baggiolini A. Varum S. Mateos J. M. Bettosini D. John N. Bonalli M. (2015). Premigratory and migratory neural crest cells are multipotent in *vivo*. *Cell Stem Cell* 16 314–322. 10.1016/j.stem.2015.02.017 25748934

[B4] Barber R. P. Vaughn J. E. (1986). Differentiation of dorsal root ganglion cells with processes in their synaptic target zone of embryonic mouse spinal cord: a retrograde tracer study. *J. Neurocytol.* 15 207–218. 10.1007/bf01611657 3723148

[B5] Belmadani A. Tran P. B. Ren D. Assimacopoulos S. Grove E. A. Miller R. J. (2005). The chemokine stromal cell-derived factor-1 regulates the migration of sensory neuron progenitors. *J. Neurosci.* 25 3995–4003. 10.1523/jneurosci.4631-04.2005 15843601 PMC4461238

[B6] Betancur P. Bronner-Fraser M. Sauka-Spengler T. (2010). Assembling neural crest regulatory circuits into a gene regulatory network. *Annu. Rev. Cell Dev. Biol.* 26 581–603. 10.1146/annurev.cellbio.042308.113245 19575671 PMC4040144

[B7] Betancur P. Simoes-Costa M. Sauka-Spengler T. Bronner M. E. (2014). Expression and function of transcription factor cMyb during cranial neural crest development. *Mech. Dev.* 132 38–43. 10.1016/j.mod.2014.01.005 24509349 PMC3987950

[B8] Bronner M. E. LaBonne C. (2012). Preface: the neural crest–from stem cell formation to migration and differentiation. *Dev. Biol.* 366:1. 10.1016/j.ydbio.2012.03.011 22459578

[B9] Bronner-Fraser M. Garcia-Castro M. (2008). Manipulations of neural crest cells or their migratory pathways. *Methods Cell Biol.* 87 75–96. 10.1016/s0091-679x(08)00204-5 18485292

[B10] Bucala R. (1996). MIF rediscovered: cytokine, pituitary hormone, and glucocorticoid-induced regulator of the immune response. *FASEB J.* 10 1607–1613. 10.1096/fasebj.10.14.9002552 9002552

[B11] Bush J. O. Lan Y. Maltby K. M. Jiang R. (2002). Isolation and developmental expression analysis of Tbx22, the mouse homolog of the human X-linked cleft palate gene. *Dev. Dyn.* 225 322–326. 10.1002/dvdy.10154 12412015

[B12] Castro D. S. Martynoga B. Parras C. Ramesh V. Pacary E. Johnston C. (2011). A novel function of the proneural factor Ascl1 in progenitor proliferation identified by genome-wide characterization of its targets. *Genes Dev.* 25 930–945. 10.1101/gad.627811 21536733 PMC3084027

[B13] Chen Z. F. Behringer R. R. (1995). twist is required in head mesenchyme for cranial neural tube morphogenesis. *Genes Dev.* 9 686–699. 10.1101/gad.9.6.686 7729687

[B14] Chesney J. A. Mitchell R. A. (2015). 25 years on: a retrospective on migration inhibitory factor in tumor angiogenesis. *Mol. Med.* 21(Suppl. 1), S19–S24. 10.2119/molmed.2015.00055 26605643 PMC4661055

[B15] Chiu I. M. Barrett L. B. Williams E. K. Strochlic D. E. Lee S. Weyer A. D. (2014). Transcriptional profiling at whole population and single cell levels reveals somatosensory neuron molecular diversity. *eLife* 3:e04660. 10.7554/eLife.04660 PMC4383053 25525749

[B16] Cloutier J. F. Sahay A. Chang E. C. Tessier-Lavigne M. Dulac C. Kolodkin A. L. (2004). Differential requirements for semaphorin 3F and Slit-1 in axonal targeting, fasciculation, and segregation of olfactory sensory neuron projections. *J. Neurosci.* 24 9087–9096. 10.1523/jneurosci.2786-04.2004 15483127 PMC6730055

[B17] De Bellard M. E. Bronner-Fraser M. (2005). Neural crest migration methods in the chicken embryo. *Methods Mol. Biol.* 294 247–267. 15576917 10.1385/1-59259-860-9:247

[B18] De Bellard M. E. Ortega B. Sao S. Kim L. Herman J. Zuhdi N. (2018). Neuregulin-1 is a chemoattractant and chemokinetic molecule for trunk neural crest cells. *Dev. Dyn.* 247 888–902. 10.1002/dvdy.24625 29516589 PMC6105535

[B19] Duband J. L. Tucker G. C. Poole T. J. Vincent M. Aoyama H. Thiery J. P. (1985). How do the migratory and adhesive properties of the neural crest govern ganglia formation in the avian peripheral nervous system? *J. Cell. Biochem.* 27 189–203. 10.1002/jcb.240270302 3886676

[B20] Dyson L. Holmes A. Li A. Kulesa P. M. (2018). A chemotactic model of trunk neural crest cell migration. *Genesis* 56:e23239. 10.1002/dvg.23239 30133140

[B21] Etchevers H. C. Vincent C. Le Douarin N. M. Couly G. F. (2001). The cephalic neural crest provides pericytes and smooth muscle cells to all blood vessels of the face and forebrain. *Development* 128 1059–1068. 11245571 10.1242/dev.128.7.1059

[B22] Farinas I. Wilkinson G. A. Backus C. Reichardt L. F. Patapoutian A. (1998). Characterization of neurotrophin and Trk receptor functions in developing sensory ganglia: direct NT-3 activation of TrkB neurons *in vivo*. *Neuron* 21 325–334. 10.1016/s0896-6273(00)80542-5 9728914 PMC2711510

[B23] Fujita K. Yasui S. Shinohara T. Ito K. (2011). Interaction between NF-kappaB signaling and Notch signaling in gliogenesis of mouse mesencephalic neural crest cells. *Mech. Dev.* 128 496–509. 10.1016/j.mod.2011.09.003 21983543

[B24] Gammill L. S. Bronner-Fraser M. (2003). Neural crest specification: migrating into genomics. *Nat. Rev. Neurosci.* 4 795–805. 10.1038/nrn1219 14523379

[B25] Gammill L. S. Gonzalez C. Gu C. Bronner-Fraser M. (2006). Guidance of trunk neural crest migration requires neuropilin 2/semaphorin 3F signaling. *Development* 133 99–106. 10.1242/dev.02187 16319111

[B26] Giovannone D. Ortega B. Reyes M. El-Ghali N. Rabadi M. Sao S. (2015). Chicken trunk neural crest migration visualized with HNK1. *Acta Histochem.* 117 255–266. 10.1016/j.acthis.2015.03.002 25805416 PMC4414037

[B27] Giovannone D. Reyes M. Reyes R. Correa L. Martinez D. Ra H. (2012). Slits affect the timely migration of neural crest cells via Robo receptor. *Dev. Dyn.* 241 1274–1288. 10.1002/dvdy.23817 22689303 PMC3632352

[B28] Gitler A. D. Brown C. B. Kochilas L. Li J. Epstein J. A. (2002). Neural crest migration and mouse models of congenital heart disease. *Cold Spring Harb. Symp. Quant. Biol.* 67 57–62. 10.1101/sqb.2002.67.57 12858524

[B29] Gurok U. Steinhoff C. Lipkowitz B. Ropers H. H. Scharff C. Nuber U. A. (2004). Gene expression changes in the course of neural progenitor cell differentiation. *J. Neurosci.* 24 5982–6002. 10.1523/jneurosci.0809-04.2004 15229246 PMC6729244

[B30] Higashihori N. Buchtová M. Richman J. M. (2010). The function and regulation of TBX22 in avian frontonasal morphogenesis. *Dev. Dyn.* 239 458–473. 10.1002/dvdy.22182 20033915

[B31] Jheon A. H. Schneider R. A. (2009). The cells that fill the bill: neural crest and the evolution of craniofacial development. *J. Dent. Res.* 88 12–21. 10.1177/0022034508327757 19131312 PMC3317957

[B32] Jongbloets B. C. Pasterkamp R. J. (2014). Semaphorin signalling during development. *Development* 141 3292–3297. 10.1242/dev.105544 25139851

[B33] Kanehisa M. Goto S. (2000). KEGG: kyoto encyclopedia of genes and genomes. *Nucleic Acids Res.* 28 27–30. 10592173 10.1093/nar/28.1.27 PMC102409

[B34] Kasemeier-Kulesa J. C. Mclennan R. Romine M. H. Kulesa P. M. Lefcort F. (2010). CXCR4 controls ventral migration of sympathetic precursor cells. *J. Neurosci.* 30 13078–13088. 10.1523/JNEUROSCI.0892-10.2010 20881125 PMC6633505

[B35] Kim D. H. Kim H. R. Choi E. J. Kim D. Y. Kim K. K. Kim B. S. (2014). Neural epidermal growth factor-like like protein 2 (NELL2) promotes aggregation of embryonic carcinoma P19 cells by inducing N-Cadherin expression. *PLoS One* 9:e85898. 10.1371/journal.pone.0085898 24465772 PMC3897553

[B36] Kolde R. Kolde M. R. (2015). *Package ‘pheatmap’. R Package 1.* Available online at: http://cran.r-project.org/web/packages/pheatmap/index.html (accessed October 20, 2019).

[B37] Kolde R. Vilo J. (2015). GOsummaries: an R Package for visual functional annotation of experimental data. *F1000Res.* 4:574. 10.12688/f1000research.6925.1 26913188 PMC4743157

[B38] Kulesa P. M. McLennan R. (2015). Neural crest migration: trailblazing ahead. *F1000Prime Rep.* 7:02. 10.12703/P7-02 25705385 PMC4311270

[B39] Le Douarin N. M. (2008). Developmental patterning deciphered in avian chimeras. *Dev. Growth Differ.* 50(Suppl. 1), S11–S28. 10.1111/j.1440-169X.2008.00989.x 18430163

[B40] Le Douarin N. M. Dupin E. (2003). Multipotentiality of the neural crest. *Curr. Opin. Genet. Dev.* 13 529–536. 10.1016/j.gde.2003.08.002 14550420

[B41] Le Douarin N. M. Dupin E. (2018). The “beginnings” of the neural crest. *Dev. Biol.* 444(Suppl. 1), S3–S13. 10.1016/j.ydbio.2018.07.019 30048640

[B42] Le Lievre C. S. Le Douarin N. M. (1975). Mesenchymal derivatives of the neural crest: analysis of chimaeric quail and chick embryos. *J. Embryol. Exp. Morphol.* 34 125–154. 1185098

[B43] Leung A. W. Murdoch B. Salem A. F. Prasad M. S. Gomez G. A. Garcia-Castro M. I. (2016). WNT/beta-catenin signaling mediates human neural crest induction via a pre-neural border intermediate. *Development* 143 398–410. 10.1242/dev.130849 26839343 PMC4760313

[B44] Lim J. Thiery J. P. (2012). Epithelial-mesenchymal transitions: insights from development. *Development* 139 3471–3486. 10.1242/dev.071209 22949611

[B45] Liu G. Xu Z. Hao D. (2016). MicroRNA451 inhibits neuroblastoma proliferation, invasion and migration by targeting macrophage migration inhibitory factor. *Mol. Med. Rep.* 13 2253–2260. 10.3892/mmr.2016.4770 26783235

[B46] Liu Y. Yang F. C. Okuda T. Dong X. Zylka M. J. Chen C. L. (2008). Mechanisms of compartmentalized expression of Mrg class G-protein-coupled sensory receptors. *J. Neurosci.* 28 125–132. 10.1523/JNEUROSCI.4472-07.2008 18171930 PMC6671167

[B47] Lo L. C. Johnson J. E. Wuenschell C. W. Saito T. Anderson D. J. (1991). Mammalian achaete-scute homolog 1 is transiently expressed by spatially restricted subsets of early neuroepithelial and neural crest cells. *Genes Dev.* 5 1524–1537. 10.1101/gad.5.9.1524 1909283

[B48] Lobsiger C. S. Smith P. M. Buchstaller J. Schweitzer B. Franklin R. J. Suter U. (2001). SpL201: a conditionally immortalized Schwann cell precursor line that generates myelin. *Glia* 36 31–47. 10.1002/glia.1093 11571782

[B49] Logan C. Wingate R. J. Mckay I. J. Lumsden A. (1998). Tlx-1 and Tlx-3 homeobox gene expression in cranial sensory ganglia and hindbrain of the chick embryo: markers of patterned connectivity. *J. Neurosci.* 18 5389–5402. 10.1523/jneurosci.18-14-05389.1998 9651221 PMC6793508

[B50] Lourenco S. Teixeira V. H. Kalber T. Jose R. J. Floto R. A. Janes S. M. (2015). Macrophage Migration Inhibitory Factor–CXCR4 Is the Dominant Chemotactic Axis in Human Mesenchymal Stem Cell Recruitment to Tumors. *J. Immunol.* 194 3463–3474. 10.4049/jimmunol.1402097 25712213 PMC4374168

[B51] Lu M. Zhang N. Ho A. D. (1992). Genomic organization of the putative human homeobox proto-oncogene HOX-11 (TCL-3) and its endogenous expression in T cells. *Oncogene* 7 1325–1330. 1352396

[B52] MacDonald J. (2019). *affycoretools: Functions Useful for Those Doing Repetitive Analyses with Affymetrix GeneChips; 2018.* Available online at: https://www.bioconductor.org/packages/release/bioc/html/affycoretools.html/ (accessed March 30, 2019).

[B53] MacDonald J. W. (2018). *Affycoretools: Functions Useful for Those Doing Repetitive Analyses with Affymetrix GeneChips. Bioconductor*. Available online at: https://rdrr.io/bioc/affycoretools/ (accessed October 20, 2019).

[B54] Massagué J. Chen Y.-G. (2000). Controlling TGF-β signaling. *Genes Dev.* 14 627–644. 10733523

[B55] McBeath R. Pirone D. M. Nelson C. M. Bhadriraju K. Chen C. S. (2004). Cell shape, cytoskeletal tension, and RhoA regulate stem cell lineage commitment. *Dev. Cell* 6 483–495. 10.1016/s1534-5807(04)00075-9 15068789

[B56] McLennan R. Schumacher L. J. Morrison J. A. Teddy J. M. Ridenour D. A. Box A. C. (2015). Neural crest migration is driven by a few trailblazer cells with a unique molecular signature narrowly confined to the invasive front. *Development* 142 2014–2025. 10.1242/dev.117507 25977364

[B57] Menon S. Gupton S. L. (2016). Building blocks of functioning brain: cytoskeletal dynamics in neuronal development. *Int. Rev. Cell Mol. Biol.* 322 183–245. 10.1016/bs.ircmb.2015.10.002 26940519 PMC4809367

[B58] Morikawa Y. Zehir A. Maska E. Deng C. Schneider M. D. Mishina Y. (2009). BMP signaling regulates sympathetic nervous system development through Smad4-dependent and -independent pathways. *Development* 136 3575–3584. 10.1242/dev.038133 19793887 PMC2761108

[B59] Morrison J. A. Mclennan R. Wolfe L. A. Gogol M. M. Meier S. Mckinney M. C. (2017). Single-cell transcriptome analysis of avian neural crest migration reveals signatures of invasion and molecular transitions. *eLife* 6:e28415. 10.7554/eLife.28415 29199959 PMC5728719

[B60] Mu X. Silos-Santiago I. Carroll S. L. Snider W. D. (1993). Neurotrophin receptor genes are expressed in distinct patterns in developing dorsal root ganglia. *J. Neurosci.* 13 4029–4041. 10.1523/jneurosci.13-09-04029.1993 8366358 PMC6576438

[B61] Muinonen-Martin A. J. Veltman D. M. Kalna G. Insall R. H. (2010). An improved chamber for direct visualisation of chemotaxis. *PLoS One* 5:e15309. 10.1371/journal.pone.0015309 21179457 PMC3001854

[B62] Nelson B. R. Claes K. Todd V. Chaverra M. Lefcort F. (2004). NELL2 promotes motor and sensory neuron differentiation and stimulates mitogenesis in DRG in vivo. *Dev. Biol.* 270 322–335. 10.1016/j.ydbio.2004.03.004 15183717

[B63] Neuhuber B. Gallo G. Howard L. Kostura L. Mackay A. Fischer I. (2004). Reevaluation of in vitro differentiation protocols for bone marrow stromal cells: disruption of actin cytoskeleton induces rapid morphological changes and mimics neuronal phenotype. *J. Neurosci. Res.* 77 192–204. 10.1002/jnr.20147 15211586

[B64] Nishio Y. Minami A. Kato H. Kaneda K. Nishihira J. (1999). Identification of macrophage migration inhibitory factor (MIF) in rat peripheral nerves: its possible involvement in nerve regeneration. *Biochim. Biophys. Acta* 1453 74–82. 10.1016/s0925-4439(98)00086-6 9989247

[B65] Nishio Y. Nishihira J. Ishibashi T. Kato H. Minami A. (2002). Role of macrophage migration inhibitory factor (MIF) in peripheral nerve regeneration: anti-MIF antibody induces delay of nerve regeneration and the apoptosis of Schwann cells. *Mol. Med.* 8 509–520. 10.1007/bf03402160 12456989 PMC2040017

[B66] Park N. I. Guilhamon P. Desai K. Mcadam R. F. Langille E. O’connor M. (2017). ASCL1 reorganizes chromatin to direct neuronal fate and suppress tumorigenicity of glioblastoma stem cells. *Cell Stem Cell* 21 209–224.e7. 10.1016/j.stem.2017.06.004 28712938

[B67] Pattyn A. Morin X. Cremer H. Goridis C. Brunet J. F. (1999). The homeobox gene Phox2b is essential for the development of autonomic neural crest derivatives. *Nature* 399 366–370. 10.1038/20700 10360575

[B68] R Core Team (2018). *R Foundation for Statistical Computing.* Vienna: R: A language and environment for statistical computing.

[B69] Raible D. W. Ungos J. M. (2006). Specification of sensory neuron cell fate from the neural crest. *Adv. Exp. Med. Biol.* 589 170–180. 10.1007/978-0-387-46954-6_10 17076281

[B70] Rogers C. D. Saxena A. Bronner M. E. (2013). Sip1 mediates an E-cadherin-to-N-cadherin switch during cranial neural crest EMT. *J. Cell Biol.* 203 835–847. 10.1083/jcb.201305050 24297751 PMC3857483

[B71] Rothman T. P. Le Douarin N. M. Fontaine-Perus J. C. Gershon M. D. (1990). Developmental potential of neural crest-derived cells migrating from segments of developing quail bowel back-grafted into younger chick host embryos. *Development* 109 411–423. 2401204 10.1242/dev.109.2.411

[B72] Rothstein M. Bhattacharya D. Simoes-Costa M. (2018). The molecular basis of neural crest axial identity. *Dev. Biol.* 444(Suppl. 1), S170–S180. 10.1016/j.ydbio.2018.07.026 30071217 PMC6355384

[B73] Rudel C. Rohrer H. (1992). Analysis of glia cell differentiation in the developing chick peripheral nervous system: sensory and sympathetic satellite cells express different cell surface antigens. *Development* 115 519–526. 1425337 10.1242/dev.115.2.519

[B74] Saito D. Takase Y. Murai H. Takahashi Y. (2012). The dorsal aorta initiates a molecular cascade that instructs sympatho-adrenal specification. *Science* 336 1578–1581. 10.1126/science.1222369 22723422

[B75] Samavarchi-Tehrani P. Golipour A. David L. Sung H.-K. Beyer T. A. Datti A. (2010). Functional Genomics Reveals a BMP-Driven Mesenchymal-to-Epithelial Transition in the Initiation of Somatic Cell Reprogramming. *Cell Stem Cell* 7 64–77. 10.1016/j.stem.2010.04.015 20621051

[B76] Sauka-Spengler T. Bronner-Fraser M. (2006). Development and evolution of the migratory neural crest: a gene regulatory perspective. *Curr. Opin. Genet. Dev.* 16 360–366. 10.1016/j.gde.2006.06.006 16793256

[B77] Simkin J. E. Zhang D. Ighaniyan S. Newgreen D. F. (2014). Parameters affecting efficiency of in ovo electroporation of the avian neural tube and crest. *Dev. Dyn.* 243 1440–1447. 10.1002/dvdy.24163 25044826

[B78] Simões-Costa M. Bronner M. E. (2015). Establishing neural crest identity: a gene regulatory recipe. *Development* 142 242–257. 10.1242/dev.105445 25564621 PMC4302844

[B79] Simoes-Costa M. Bronner M. E. (2016). Reprogramming of avian neural crest axial identity and cell fate. *Science* 352 1570–1573. 10.1126/science.aaf2729 27339986 PMC5100669

[B80] Simoes-Costa M. Tan-Cabugao J. Antoshechkin I. Sauka-Spengler T. Bronner M. E. (2014). Transcriptome analysis reveals novel players in the cranial neural crest gene regulatory network. *Genome Res.* 24 281–290. 10.1101/gr.161182.113 24389048 PMC3912418

[B81] Sinnberg T. Levesque M. P. Krochmann J. Cheng P. F. Ikenberg K. Meraz-Torres F. (2018). Wnt-signaling enhances neural crest migration of melanoma cells and induces an invasive phenotype. *Mol. Cancer* 17:59. 10.1186/s12943-018-0773-5 29454361 PMC5816360

[B82] Stanke M. Junghans D. Geissen M. Goridis C. Ernsberger U. Rohrer H. (1999). The Phox2 homeodomain proteins are sufficient to promote the development of sympathetic neurons. *Development* 126 4087–4094. 10457017 10.1242/dev.126.18.4087

[B83] Strobl-Mazzulla P. H. Bronner M. E. (2012). Epithelial to mesenchymal transition: new and old insights from the classical neural crest model. *Semin. Cancer Biol.* 22 411–416. 10.1016/j.semcancer.2012.04.008 22575214 PMC3435443

[B84] Szabó A. Mayor R. (2018). Mechanisms of neural crest migration. *Annu. Rev. Genet.* 52 43–63. 10.1146/annurev-genet-120417-031559 30476447

[B85] Takahashi K. Nuckolls G. H. Takahashi I. Nonaka K. Nagata M. Ikura T. (2001). Msx2 is a repressor of chondrogenic differentiation in migratory cranial neural crest cells. *Dev. Dyn.* 222 252–262. 10.1002/dvdy.1185 11668602

[B86] Takaishi M. Tarutani M. Takeda J. Sano S. (2016). Mesenchymal to epithelial transition induced by reprogramming factors attenuates the malignancy of cancer cells. *PLoS One* 11:e0156904. 10.1371/journal.pone.0156904 27258152 PMC4892607

[B87] Tang W. Li Y. Gandhi S. Bronner M. (2019). Multiplex clonal analysis in the chick embryo using retrovirally-mediated combinatorial labeling. *Dev. Biol.* 450 1–8. 10.1016/j.ydbio.2019.03.007 30885528 PMC6487888

[B88] Teillet M. A. Kalcheim C. Le Douarin N. M. (1987). Formation of the dorsal root ganglia in the avian embryo: segmental origin and migratory behavior of neural crest progenitor cells. *Dev. Biol.* 120 329–347. 10.1016/0012-1606(87)90236-3 3549390

[B89] Terheyden-Keighley D. Zhang X. Brand-Saberi B. Theiss C. (2018). CXCR4/SDF1 signalling promotes sensory neuron clustering *in vitro*. *Biol. Open* 7:bio035568. 10.1242/bio.035568 30135081 PMC6176946

[B90] Thiery J. P. Acloque H. Huang R. Y. J. Nieto M. A. (2009). Epithelial-mesenchymal transitions in development and disease. *Cell* 139 871–890. 10.1016/j.cell.2009.11.007 19945376

[B91] Tsarovina K. Reiff T. Stubbusch J. Kurek D. Grosveld F. G. Parlato R. (2010). The Gata3 transcription factor is required for the survival of embryonic and adult sympathetic neurons. *J. Neurosci.* 30 10833–10843. 10.1523/JNEUROSCI.0175-10.2010 20702712 PMC6634692

[B92] Vujicic M. Saksida T. Despotovic S. Bajic S. S. Laliæ I. Koprivica I. (2018). The role of macrophage migration inhibitory factor in the function of intestinal barrier. *Sci. Rep.* 8 6337. 10.1038/s41598-018-24706-3 29679061 PMC5910418

[B93] Walheim C. C. Zanin J. P. De Bellard M. E. (2012). Analysis of trunk neural crest cell migration using a modified Zigmond chamber assay. *J. Vis. Exp*. 59:3330. 10.3791/3330 22297254 PMC3462580

[B94] Watanabe K. Liu Y. Noguchi S. Murray M. Chang J.-C. Kishima M. (2019). OVOL2 induces mesenchymal-to-epithelial transition in fibroblasts and enhances cell-state reprogramming towards epithelial lineages. *Sci. Rep.* 9:6490. 10.1038/s41598-019-43021-z 31019211 PMC6482152

[B95] Wells A. Yates C. Shepard C. R. (2008). E-cadherin as an indicator of mesenchymal to epithelial reverting transitions during the metastatic seeding of disseminated carcinomas. *Clin. Exp. Metastasis* 25 621–628. 10.1007/s10585-008-9167-1 18600305 PMC2929356

[B96] Williams R. M. Candido-Ferreira I. Repapi E. Gavriouchkina D. Senanayake U. Ling I. T. C. (2019). Reconstruction of the global neural crest gene regulatory network *in vivo*. *Dev. Cell* 51 255–276.e7. 10.1016/j.devcel.2019.10.003 31639368 PMC6838682

[B97] Wolfstetter G. Dahlitz I. Pfeifer K. Töpfer U. Alt J. A. Pfeifer D. C. (2019). Characterization of *Drosophila Nidogen/entactin* reveals roles in basement membrane stability, barrier function and nervous system patterning. *Development* 146:dev168948. 10.1242/dev.168948 30567930

[B98] Wurdak H. Ittner L. M. Lang K. S. Leveen P. Suter U. Fischer J. A. (2005). Inactivation of TGFbeta signaling in neural crest stem cells leads to multiple defects reminiscent of DiGeorge syndrome. *Genes Dev.* 19 530–535. 10.1101/gad.317405 15741317 PMC551573

[B99] You Z.-H. Lei Y.-K. Zhu L. Xia J. Wang B. (2013). Prediction of protein-protein interactions from amino acid sequences with ensemble extreme learning machines and principal component analysis. *BMC Bioinformatics* 14:S10. 10.1186/1471-2105-14-S8-S10 23815620 PMC3654889

[B100] Young H. M. Hearn C. J. Ciampoli D. Southwell B. R. Brunet J. F. Newgreen D. F. (1998). A single rostrocaudal colonization of the rodent intestine by enteric neuron precursors is revealed by the expression of Phox2b, Ret, and p75 and by explants grown under the kidney capsule or in organ culture. *Dev. Biol.* 202 67–84. 10.1006/dbio.1998.8987 9758704

[B101] Zhang Y. Hu W. (2012). NFκB signaling regulates embryonic and adult neurogenesis. *Front. Biol.* 7: 10.1007/s11515-012-1233-z. 10.1007/s11515-012-1233-z 24324484 PMC3855406

[B102] Zhou Q. Yan X. Gershan J. Orentas R. J. Johnson B. D. (2008). Expression of macrophage migration inhibitory factor by neuroblastoma leads to the inhibition of antitumor T cell reactivity *in vivo*. *J. Immunol.* 181 1877–1886. 10.4049/jimmunol.181.3.1877 18641325 PMC3804024

