# Supplementary figures and images for: Molecular Events Controlling Cessation of Trunk Neural Crest Migration and Onset of Differentiation
Source: Front Cell Dev Biol. 2020 Apr 2;8:199. doi: 10.3389/fcell.2020.00199 (PMC7147452; doi:10.3389/fcell.2020.00199)

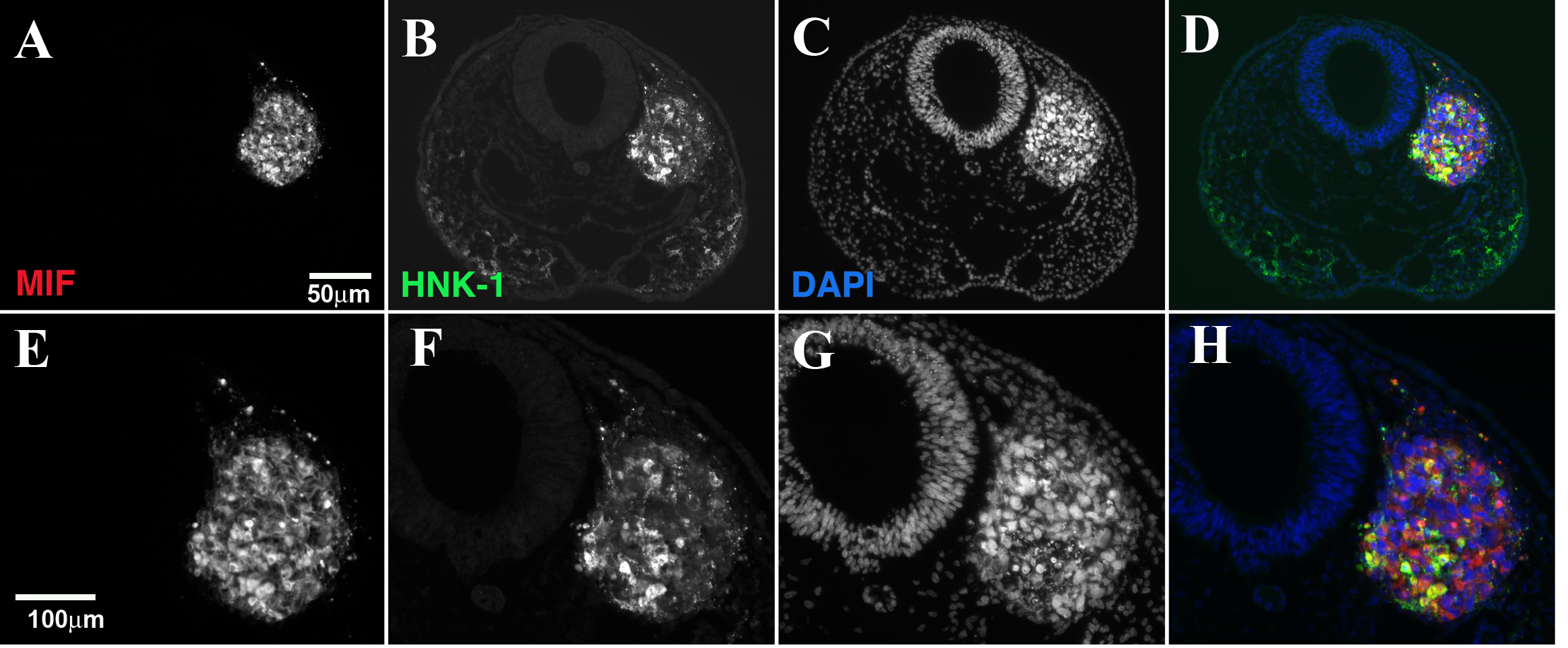

Supplement: FIGURE S1 — Exogenous MIF attracts tNCC in vivo. (A–D) Sections in an injected embryo shows HNK1 (green) and, DiI U205 MIF-expressing cells (red), in the head of a HH10 embryo. (E–H) A higher magnification image in (A–D) showing cranial NCC enveloping MIF expressing U205 cell. DAPI labels nuclei in blue. Scale Bars are for microns. [file Image_1.TIF]

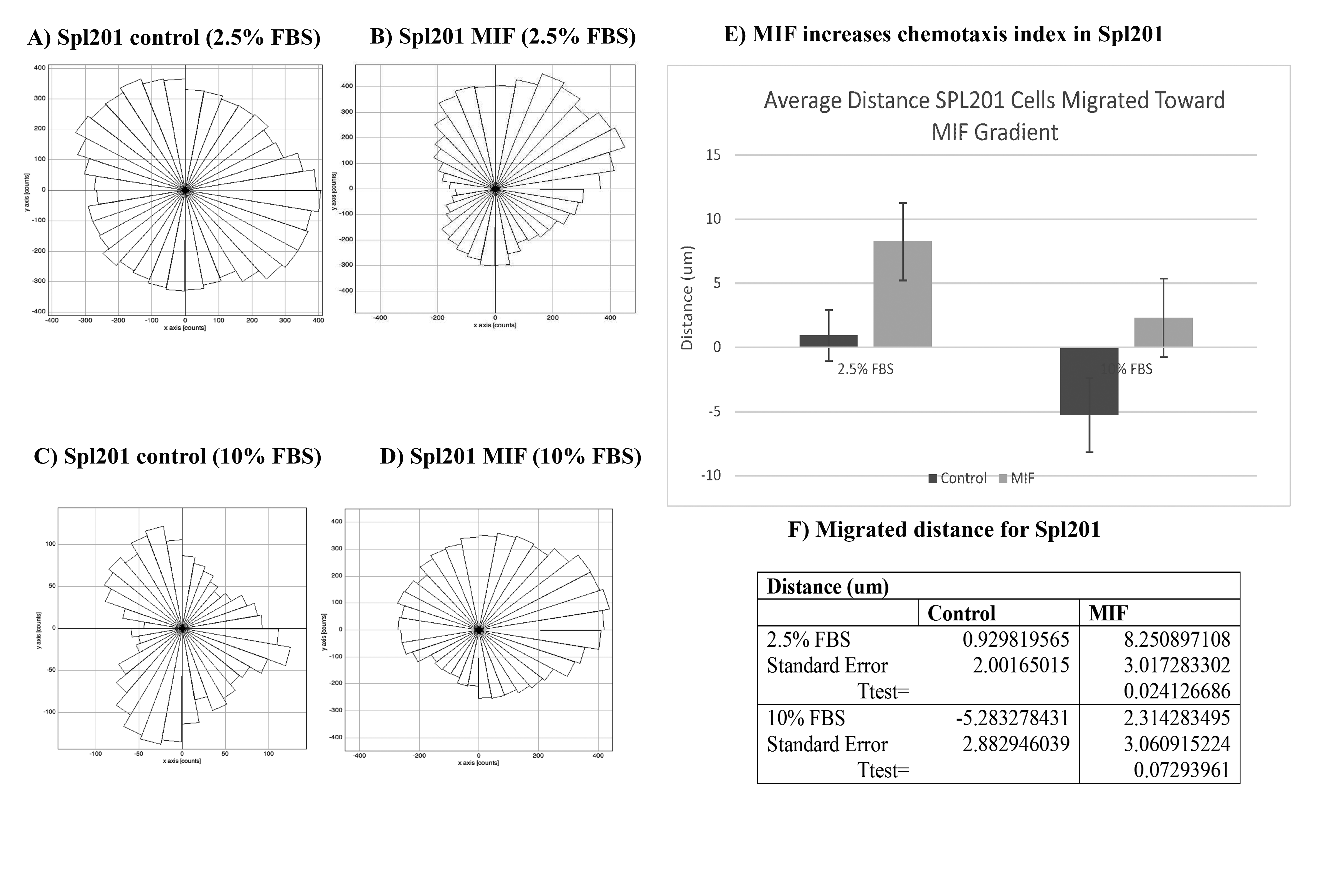

Supplement: FIGURE S2 — MIF attracts Spl201 in vitro. The NCC cell line Spl201 was seeded into Ibidi chambers in the presence (B,D) or absence of MIF (A,C). Graph shows a rosette presentation of all the tracked paths. MIF was in the top chamber were we observe most of the Spl201 cells migrated in contrast to control that there are equal numbers of tracks in top and bottom portion of the graph. (E) Graph of chemotaxis index from (A–D) experiments. MIF index is significantly positive (attractant) compared with control media. (F) Migrated distance of Spl201 in presence or absence of MIF in the media. [file Image_2.TIF]
