# Supplementary material for: Molecular Events Controlling Cessation of Trunk Neural Crest Migration and Onset of Differentiation
Source: Front Cell Dev Biol. 2020 Apr 2;8:199. doi: 10.3389/fcell.2020.00199 (PMC7147452; doi:10.3389/fcell.2020.00199)
Supplement: TABLE S3 — Q-PCR primers. [file Table_3.docx]

**Additional files**

**Table S3: Q-PCR primers**

| Gene name | Forward primers | Reverse Primers |
| --- | --- | --- |
| CRABP-1 | caacttcaaaatcggggaga | tcatcagcaccaaaggtcaa |
| ADAMTSL1 | agccccaaaaagatccagtt | aactctggcattctgcctgt |
| TLX3 | aggtgaagacgtggttccag | atcgactcgttgagcgactt |
| ADCYAP1 | gctaagctggaggaggaggt | cggccgctaagtatttcttg |
| MAB21L1 | gggacgtggtaaagatggtg | ccttggagagcaggttgaag |
| CFC1 | catttaggaaaaggccgtga | tctgtaatcccggtgaaagg |
| STMN2 | aacatgagcgagaggtgctt | gcatgcctctccttttcttg |
| SANAP25 | gaatgctgcagcttgttgaa | aacgccatcctgattattgc |
| Semilar to ADAM12 | gagcccagcgagatcttcta | gtggcacatggtgtgtcttc |
| TBX22 | tctcctctcaacccactgct | agcttgggtacatcgaccac |
| FGFR2 | gtggcagtgaagatgctgaa | acgtgcaggataccaagtcc |
| Osteopontin | cacagaatgacctggcttca | gccatatgccacactgtcac |
| OSF2 | gatcatgggtggagctgtct | caatgacttgcttggcagaa |
| RARB | gcctgcctggacatactgat | tgcatctgagttcggttcag |
| FZD7 | tggaacgcttctctgaggat | gccatgccgaagaagtagag |
| SEMA3C | gaggagtcgtaggcaagacg | tgtcttggacagagcgaatg |
