# Supplementary material for: Molecular Events Controlling Cessation of Trunk Neural Crest Migration and Onset of Differentiation
Source: Front Cell Dev Biol. 2020 Apr 2;8:199. doi: 10.3389/fcell.2020.00199 (PMC7147452; doi:10.3389/fcell.2020.00199)
Supplement: TABLE S4 — Key resources table. [file Table_4.docx]

**KEY RESOURCES TABLE**

| **REAGENT or RESOURCE** | **SOURCE** | | **IDENTIFIER** |
| --- | --- | --- | --- |
| **Antibodies** | | | |
| Mouse HNK-1 IgM | DSHB | | Cat#3H5 |
| TuJ1 clone | COVANCE | | TUJ1 |
| Alexa 488 anti-mouse IgM | ThermoFisher | | Cat# A-21042 |
| Alexa 488 anti-rabbit IgG | ThermoFisher | | Cat#A-11034 |
| Alexa 594 anti-rabbit IgG | ThermoFisher | | Cat#A11037 |
| Alexa 488 anti-mouse IgM | Invitrogen | | Cat# A-21042 |
| cell tracker CM-DiI | Invitrogen | | Cat# C-7001 |
| DAPI | Molecular Probes | |  |
| **Biological Samples** | | | |
| **Chemicals, Peptides, and Recombinant Proteins** | | | |
| BSA | ThermoFisher | | Cat#BP-1600-100 |
| Permafluor | ThermoFisher | | TA-006-FM |
| **Critical Commercial Assays** | | | |
| Boyden Chamber Assay | BD | |  |
| **Experimental Models: Organisms/Strains** | | | |
| Chicken embryos (*Gallus gallus*) | AA Laboratories, Westminster, CA | |  |
| **Recombinant DNA** | | | |
| DN-erbB4 | Gabriel Corfas | | <https://doi.org/10.1016/S0896-6273(00)80346-3>. |
| pcDNA3-EGFP | Doug Golenbock,Addgene | | #13031 |
| **Software and Algorithms** | | | |
| Excel | Microsoft | | <https://products.office.com/en-us/excel> |
| GeneSpring | Agilent Technologies | | <http://genespring-support.com/support/feature-content#intro> |
| Bio-Rad CFX-96 | Bio-Rad | | <http://www.bio-rad.com/en-us/category/qpcr-analysis-software> |
| Agilent’s Feature Extraction software version 11.0.1.1 | Agilent | |  |
| Excel | Microsoft | | <https://products.office.com/en-us/excel> |
| IPA | Qiagen | | <https://www.qiagenbioinformatics.com/products/ingenuity-pathway-analysis/> |
| The **D**atabase for **A**nnotation, **V**isualization and**I**ntegrated **D**iscovery (**DAVID**) |  | | <https://david.ncifcrf.gov/> |
| ImageJ Manual Tracking | ImageJ | | <https://imagej.nih.gov/ij/plugins/track/track.html> |
| ImageJ Chemotaxis and Migration Tool | ImageJ | | <http://ibidi.com/software/chemotaxis_and_migration_tool> |
| AxioVision Rel. 4.8 | Zeiss | | <https://www.zeiss.com/microscopy/int/downloads/axiovision-downloads.html> |
| **Experimental Models: Cell Lines** | | | |
| SpL201 | | Johanna Buchstaller | GLIA 36:31–47, 2001. DOI:10.1002/glia.1093 |
| HEK293 | | ATCC | ATCC^®^ CRL-1573^™^ |
| **Recombinant DNA** | | | |
| pMES-GFP | | Marianne Bronner |  |
| pMES-MIF | | Vivian Lee |  |
